# Root‐sourced 1‐aminocyclopropane‐1‐carboxylic acid modulates development and adaptive responses to salinity in tomato

**DOI:** 10.1111/tpj.71133

**Published:** 2026-10-08

**Authors:** Jose A. Martín‐Rodríguez, Purificación A. Martínez‐Melgarejo, Jesús Guillamón, Ángela S. Prudencio, Juan J. Guerrero, Eduardo Larriba, Jose Manuel Pérez‐Pérez, Enrique Olmos, Lázaro E. Pereira Peres, Cristina Martínez‐Andújar, Francisco Pérez‐Alfocea

**Affiliations:** ^1^ Group of Plant Hormones CEBAS‐CSIC Murcia Spain; ^2^ Institute of Bioengineering Miguel Hernández University Elche Spain; ^3^ Department of Stress Biology CEBAS‐CSIC Murcia Spain; ^4^ Laboratory of Hormonal Control of Plant Development, Escola Superior de Agricultura ‘Luiz de Queiroz’ Universidade de São Paulo Piracicaba Brazil

**Keywords:** ABA metabolism, 1‐aminocyclopropane‐1‐carboxylic acid, leaf transcriptomics, plant hormones, root–shoot communication, root system architecture, *Solanum lycopersicum*, source–sink

## Abstract

To investigate whether root‐sourced ethylene‐precursor 1‐aminocyclopropane‐1‐carboxylic acid (ACC) modulates long‐term salinity responses in tomato, a commercial variety (*Solanum lycopersicum* L. Mill) was grafted onto three rootstocks differing in ACC/ethylene production: an ethylene‐overproducing mutant (*epinastic*, *epi*), a root‐specific *ACC deaminase* overexpressing transgenic line with reduced ACC availability for ethylene synthesis (*ACCD*) and the wild‐type cv. Micro‐Tom (WT). *ACCD* grafts exhibited low‐ACC concentrations, a branched root system and enhanced plant growth and yield stability under salinity, compared to *epi* and WT grafts, through modulating nutritional, metabolic, hormonal and transcriptional networks in the scion. Low root‐sourced ACC was linked to down‐ and upregulation of ABA biosynthesis (*NCED3*) and catabolism (*CYP707A1/3*), leading to reduced leaf ABA but induced signalling; activation of GA biosynthesis (*GA20ox*, *GA3ox*) and CK homeostasis (*ZOG*, *AHPT1L*, *ARR‐Bs*, *CRF2/4*); induced steroid metabolism; improved leaf P (*SPX3*, *PAP17, PEPC1*), Ca and S nutrition, and limited Na^+^ accumulation, leading to stable carbon metabolism, source–sink relations and yield under salt stress. High root‐sourced ACC in *epi* grafts mainly penalised reproductive growth but induced protective flavonoids and source activity in salinised leaves. These findings highlight the role of root‐derived ACC in modulating local and systemic responses to stress and its potential for improving crop yield stability under salinity.

## Significance Statement

Root‐derived ACC modulates tomato growth and adaptation to salinity. Grafting a commercial variety onto Micro‐Tom based rootstocks differing in ACC/ethylene production supports the conclusion that lowering root ACC enhances plant growth and yield stability through hormonal and nutritional balance, carbon metabolism and optimal source–sink relations: In contrast, high root ACC is associated with adaptive responses to salinity. Modulation of root‐to‐shoot ACC production offers new opportunities for improving crop resilience and productivity in saline environments.

## INTRODUCTION

Soil salinisation can occur naturally or from anthropogenic factors such as poor irrigation and fertiliser overuse (Guida‐Johnson et al., 2017; Velmurugan et al., 2020). Salinity reduces crop growth through osmotic stress and ionic toxicity from Na and Cl accumulation, disrupting nutrient uptake (Munns, 2002; Munns & Tester, 2008). The root system not only mediates water and nutrient acquisition but also regulates key processes under salinity, including Na exclusion and hormonal signalling. Root‐to‐shoot hormonal communication influences shoot physiology, plant growth and yield (Albacete et al., 2015; Dodd, 2005; Julkowska et al., 2017), providing a biotechnological strategy to improve crop salt tolerance through altering root hormone metabolism (Ghanem et al., 2011; Martínez‐Andújar et al., 2021; Pérez‐Alfocea et al., 2010).

The root system architecture (RSA) varies significantly in response to salt depending on plant species and salt concentration, although in general, salinity inhibits both primary root (PR) and lateral root (LR) growth (Zou et al., 2022). Furthermore, several plant hormones regulate plant responses to salt stress by modulating RSA. Ethylene signalling is involved in RSA changes and whole‐plant adaptation to nutrient deficiency and has been related to reduced PR (Jung et al., 2009) and LR growth (Lewis et al., 2011). Interestingly, exogenous application of the ethylene‐precursor ACC reduces both PR and LR (Duan et al., 2013; Kawa et al., 2020). In contrast, the application of ethylene inhibitors induces PR elongation and increases both the number and size of LR (Belimov et al., 2022). Moreover, ethylene interacts with other hormones such as ABA in shaping RSA, while excessive ethylene usually has a negative impact on root growth (Liu et al., 2024). The role of ethylene and its precursor ACC in the response to abiotic stresses, particularly salinity, remains controversial. In tomato and other species, salinity increases ethylene and ACC levels (Riyazuddin et al., 2020), which can exacerbate stress responses by promoting oxidative damage and leaf senescence (Albacete et al., 2008; Cao et al., 2008; Ghanem et al., 2008). Conversely, in *Arabidopsis*, exogenous application of ethylene or ACC eliminates ROS, increasing tolerance to salinity (Peng et al., 2014). Several works have shown that ethylene plays an important role in the regulation of nutrient uptake and nutrient/Na homeostasis under salinity (Tao et al., 2015). Reducing ACC content through the action of growth‐promoting bacteria (PGPB) that produce ACC deaminase (*ACCD*), which breaks ACC into α‐ketobutyrate and ammonia, decreases ethylene production and mitigates its potential adverse effects under salt stress (Naing et al., 2021; Win et al., 2018). Moreover, transgenic plants overexpressing the *ACCD* gene have been generated in the past decades, demonstrating improved tolerance to multiple abiotic stresses (Jung et al., 2018; Zhang et al., 2008).

Ethylene interacts with multiple phytohormones during abiotic stress responses. Under salinity, ABA regulates several ethylene biosynthetic genes (Tao et al., 2015). Salinity also down‐regulates *EIN2*, a key component of ethylene signalling, resulting in increased ABA production (Wang et al., 2007). Furthermore, under salinity, ethylene stabilises DELLA proteins, repressors of GAs signalling, causing growth arrest (Bhardwaj et al., 2022; Iqbal et al., 2012). Finally, an antagonistic interaction between ethylene and CKs under salt stress is characterised by decreased CK levels (Riyazuddin et al., 2020).

Tomato (*Solanum lycopersicum* L.) is one of the most important crops worldwide (5 million cultivated ha) (FAOSTAT, 2023). Many of the cultivated areas are affected by salinity, such as the South‐Eastern Spain, where grafting onto vigorous rootstocks is a common commercial practice to alleviate the effect of biotic and abiotic stresses on yield without compromising fruit quality (Bletsos & Olympios, 2008; Martínez‐Andújar et al., 2018) and provides a model to study how root‐targeted hormonal regulation affects whole‐plant responses (Albacete et al., 2015; Ghanem et al., 2011; Pérez‐Alfocea et al., 2010; Pérez‐Alfocea et al., 2021). Previous studies in tomato have demonstrated that ACC exhibits a dynamic increase in roots, xylem sap and shoots in response to salinity and has been correlated with the onset of salt‐induced leaf senescence (Ghanem et al., 2008) and growth reduction (Albacete et al., 2008). Moreover, increased tolerance to abiotic stresses, through rootstock genetic variability or root‐specific alteration in hormone biosynthesis (e.g. CKs and ABA), has been consistently associated with reduced ACC evolution (Albacete et al., 2009; Albacete et al., 2010; Albacete et al., 2015; Albacete, Cantero‐Navarro, et al., 2014; Albacete, Martínez‐Andújar, & Pérez‐Alfocea, 2014; Martínez‐Andújar et al., 2016; Martínez‐Andújar et al., 2017; Martínez‐Andújar et al., 2021). Indeed, an ethylene‐independent role for ACC signalling in responses to stress through influencing processes such root and shoot growth, cell wall flexibility and stomatal development (Mou et al., 2025; Tsang et al., 2011; Vanderstraeten et al., 2019; Xu et al., 2008; Yin et al., 2019) cannot be discarded. Therefore, through a root‐specific functional analysis, this study aims to (i) investigate the long‐term (whole crop cycle) impact of modulated root‐sourced ACC/ethylene production on plant physiology and yield under salinity in an important crop species, as the tomato and (ii) provide new insights into the underlying local and systemic mechanisms.

## RESULTS

### Plant growth and yield

Under optimal conditions, shoot fresh weight (SFW) and total plant biomass (TB) did not differ among graft combinations. After 12 weeks of saline irrigation, SFW decreased in all plants; most markedly in the *epinastic* (*epi*) grafted plants. Other shoot vigour indicators such as stem diameter (SD), leaf fresh weight (LFW) and leaf area (LA) were also reduced by salinity, but to a lower extent in the plants grafted onto *ACCD* rootstocks (Table 1). Indeed, LFW was the most differential parameter in *ACCD*‐grafted plants under salinity compared to other graft combinations, suggesting differences in leaf water content and thickness. Contrary to the aerial part, salinity increased root fresh weight (RFW), but more significantly in wild‐type (WT) and *ACCD* than in *epi* grafts (Table 1; Figure 1C). *Epi* and WT grafts registered the highest and lowest yield values under control conditions, respectively, but no significant differences were observed among graft combinations. Yield of *ACCD* grafts was unaffected by salinity and produced 55% and 36% more than WT and *epi* plants under stress, respectively. This superior yield performance in salinised *ACCD* grafts compared to other rootstocks was attributed to the stability of both fruit number (FN) and fruit weight (FW) relative to control conditions. *Epi* grafts registered the highest FW values under control conditions but were the most affected by salinity, while the plants grafted onto WT rootstocks registered the lowest FN and the shortest trusses, resulting in a lower fruit per truss ratio in both conditions (Table 1). Notably, the presence of multiple trusses under salt stress conditions was observed exclusively in *ACCD* grafts (Table 1). Overall, *ACCD* rootstocks were the most productive and stable in vegetative and reproductive traits in whatever conditions. However, on average of those traits, WT and *epi* were similarly affected by salinity (40%), but while WT was more affected in vegetative traits (60% in LA and LFW), *epi* grafts were more affected in reproductive ones (45% in FW). Although the tomato variety Unidarkwin F1 used in this study is self‐pollinated, the experiment was part of a bigger one in which managed bumblebees (*Bombus terrestris*) were used at commercial scale to ensure pollination in all plants. In order to relate pollination activity with flower analyses, observations of stamen marks during flowering distinguished non‐visited flowers (no mark) from those visited by pollinators (brown marks). Albeit non‐significant, *ACCD*‐grafted plants exhibited about 20% more flowers visited by bumblebees (FVB) compared to other grafts (Table 1), suggesting a higher preference of bumblebees for those plants, which could potentially influence fruit setting and development under stress.

**Table 1:** Biomass, production, photosynthesis and chlorophyll parameters

		Control			Salt			*P* (ANOVA)		
		UD/WT	UD/*epi*	UD/*ACCD*	UD/WT	UD/*epi*	UD/*ACCD*	G	T	GXT
Biomass	SFW (g·plant^−1^)	807.25 ± 133.26^ab^	732.77 ± 103.06^ab^	889.88 ± 93.33^a^	526.67 ± 212.97^bc^	426.67 ± 38.59^c^	642.16 ± 89.18^b^	0.350	0.012*	0.973
	RFW (g·plant^−1^)	45.22 ± 2.31^c^	50.77 ± 2.34^bc^	63.88 ± 7.54^b^	67.63 ± 25.20^ab^	69.47 ± 20.72^ab^	92.93 ± 8.91^a^	0.162	0.030*	0.913
	TB (g·plant^−1^)	1976.71 ± 101.34^ab^	2214.45 ± 77.25^a^	2360.08 ± 243.03^a^	1354.21 ± 404.34^b^	1350.25 ± 97.28^b^	2066.94 ± 251.69^a^	0.052	0.027*	0.412
	SD (mm·plant^−1^)	12.74 ± 0.42^b^	15.16 ± 0.58^ab^	16.30 ± 0.31^a^	8.97 ± 1.35^c^	9.67 ± 0.87^c^	12.91 ± 0.98^b^	<0.001***	<0.001***	0.428
	LFW (g)	25.70 ± 1.85^a^	23.13 ± 5.05^a^	31.70 ± 2.20^a^	10.87 ± 3.55^b^	13.00 ± 1.04^b^	27.95 ± 3.67^a^	0.005**	0.003**	0.300
	LA (mm^2^)	407.59 ± 45.13^a^	357.42 ± 63.68^ab^	440.20 ± 19.24^a^	159.29 ± 41.69^cd^	223.41 ± 14.05^c^	324.33 ± 46.29^bc^	0.028*	<0.001***	0.295
Production	Yield (g·plant^−1^)	1018.54 ± 70.13^ab^	1430.92 ± 27.93^a^	1406.33 ± 157.61^a^	599.38 ± 157.78^b^	854.12 ± 78.47^b^	1331.85 ± 222.34^a^	0.004**	0.008**	0.217
	FN (N° fruit·plant^−1^)	95.25 ± 3.82^bc^	108.78 ± 12.62^bc^	120.00 ± 11.98^ab^	78.50 ± 5.50^c^	125.33 ± 5.84^ab^	146.28 ± 14.80^a^	<0.001***	0.534	0.134
	FW (g·plant^−1^)	9.58 ± 0.31^b^	12.07 ± 0.68^a^	10.36 ± 0.50^ab^	7.42 ± 1.24^c^	6.67 ± 0.33^c^	8.48 ± 0.74^bc^	0.292	<0.001***	0.015*
	Fruits/truss	9.16 ± 0.49^c^	11.57 ± 0.93^ab^	13.53 ± 0.48^a^	9.95 ± 1.28^bc^	11.86 ± 0.28^ab^	13.18 ± 0.88^a^	<0.001***	0.730	0.804
	Truss length (cm)	12.64 ± 0.06^c^	17.38 ± 1.71^ab^	19.36 ± 0.69^a^	13.52 ± 2.67^bc^	14.80 ± 0.39^bc^	18.60 ± 1.14^ab^	<0.001***	0.047*	0.724
	% multiple trusses	0.00 ± 0.00^b^	5.36 ± 2.85^ab^	0.00 ± 0.00^b^	0.00 ± 0.00^b^	0.00 ± 0.00^b^	7.14 ± 4.50^a^	0.238	0.738	0.019*
	%FVB	53.33 ± 4.71^b^	59.13 ± 0.56^ab^	61.80 ± 3.23^ab^	56.81 ± 13.70^ab^	59.73 ± 2.49^ab^	72.05 ± 3.39^a^	0.167	0.352	0.718
Photosynthesis	A_N_ (μmol·m^−2^·s^−1^)	7.96 ± 0.53^ab^	8.91 ± 1.45^ab^	10.04 ± 1.14^ab^	6.95 ± 1.47^b^	8.63 ± 0.91^ab^	10.3 ± 1.06^a^	0.032*	0.429	0.413
	g_S_ (mmol m^−2^ s^−1^)	147.83 ± 17.96^ab^	120.44 ± 9.42^bc^	177.00 ± 9.42^a^	72.67 ± 18.28^d^	100.92 ± 20.60^cd^	141.67 ± 26.33^abc^	0.167	0.001**	0.382
	E (nmol·m^−2^·s^−2^)	3.48 ± 0.49^ab^	3.03 ± 0.22^ab^	4.02 ± 0.40^a^	1.82 ± 0.33^c^	2.73 ± 0.47^bc^	3.28 ± 0.30^ab^	0.176	0.004**	0.425
	Fv/Fm	0.77 ± 0.01^ab^	0.77 ± 0.01^ab^	0.80 ± 0.01^a^	0.74 ± 0.02^b^	0.77 ± 0.01^ab^	0.77 ± 0.01^ab^	0.035*	0.045*	0.482
	SPAD	53.53 ± 2.72^ab^	69.23 ± 2.48^a^	68.38 ± 2.54^a^	44.00 ± 8.47^b^	41.63 ± 5.07^b^	51.98 ± 3.94^ab^	0.065	<0.001***	0.219

Biomass parameters (Shoot fresh weight, SFW; root fresh weight, RFW; total biomass, TB; stem diameter, SD; leaf fresh weight, LFW; leaf area, LA), yield‐related parameters (Total yield, Yield; fruit number, FN; fruit weight, FW; truss length, TL; % multiple trusses; % flowers visited by bumblebees, %FVB), photosynthesis‐related parameters (Photosynthesis rate, A_N_; stomatal conductance, g_S_; evapotranspiration, E), and chlorophyll‐related parameters (maximum quantum yield of open photosystem II, Fv/Fm; chlorophyll dynamics with soil plant analytical division, SPAD) of the tomato cv UniDarkwin grafted onto MT (UD/WT), *epi* (high ethylene production, UD/*epi*) and *ACCD* (low ethylene production, UD/*ACCD*) rootstocks, after 14 weeks of cultivation under control and salt (75 mM NaCl) treatments for biomass‐ and yield‐related parameters, and after 10 weeks of treatment for photosynthesis‐ and chlorophyll‐related parameters. Values indicate mean ± SE. Different letters indicate statistically significant differences among graft combinations (*n* = 6 for yield and biomass related parameters; *n* = 3 for gas exchange and chlorophyll‐related parameters, 3 readings per plant; *P* < 0.05) within and between treatments. Results of two‐way ANOVA (p values reported) for genotype (G), treatment (T) and their interaction (G × T) are indicated on the right side of the Table. *, ** and *** indicate statistically significant difference at *P* < 0.05, *P* < 0.01 and *P* < 0.001, respectively.

**Figure 1:**
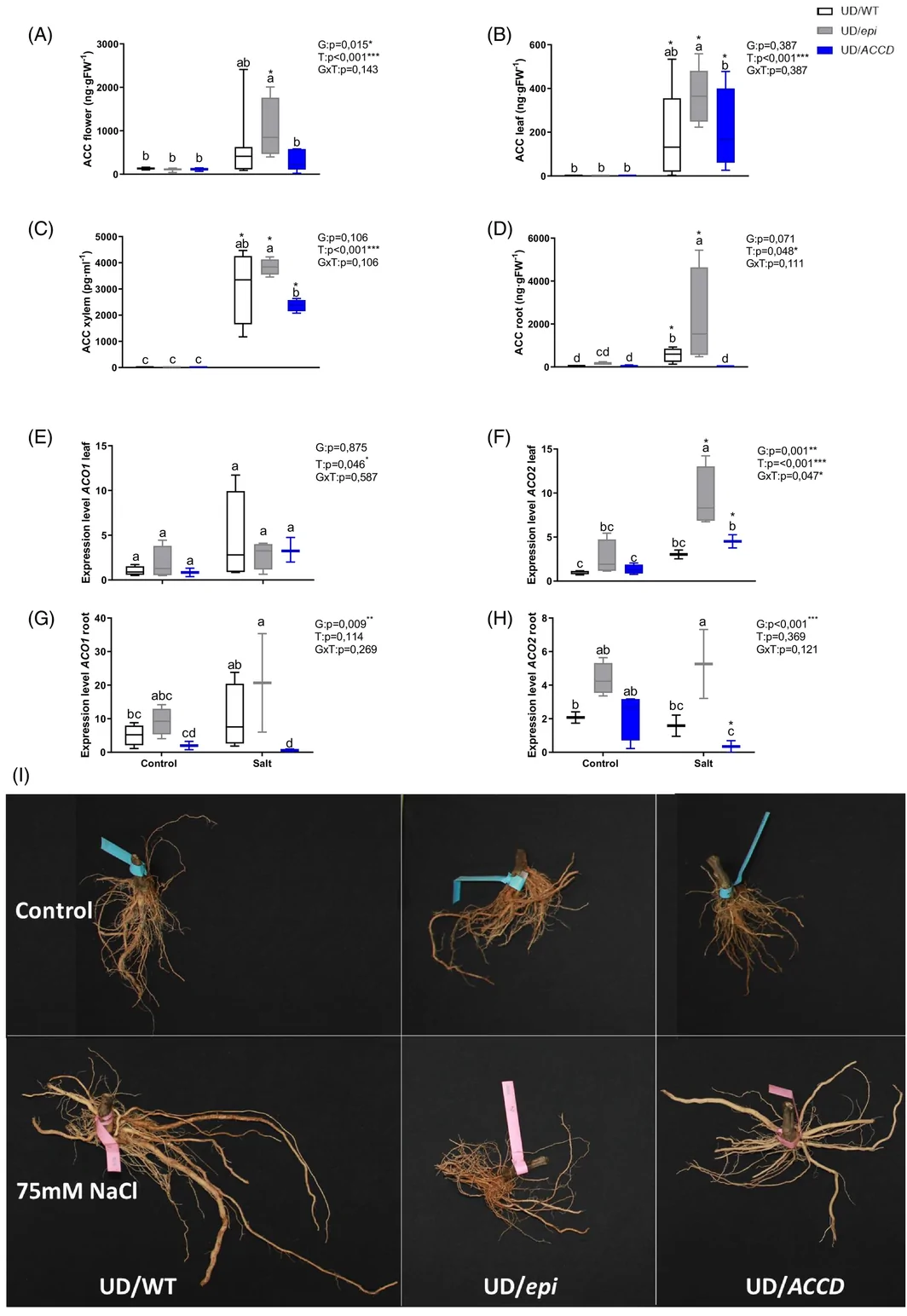
Effects of salt stress and rootstock genotype on ACC production, ethylene signalling and root morphology. 1‐aminocyclopropane‐1‐carboxylic acid (ACC) content in (A) flowers, (B) leaves, (C) xylem and (D) roots, and (E–H) expression of ethylene‐related genes 1‐aminocyclopropane‐1‐carboxylate oxidase 1 (*ACO1*, Solyc07g049530) and 2 (*ACO2*, Solyc12g005940) in roots and leaves, as determined by qRT‐PCR, of the tomato cv UniDarkwin (UD) grafted onto MT (UD/WT), epi (high ethylene production, UD/*epi*) and *ACCD* (low ethylene production, UD/*ACCD*) rootstocks after 14 weeks growing under control and salt (75 mM NaCl) treatment. Bars indicate mean values ± SE. Different letters indicate significant differences among graft combinations (*n* = 4 for qPCR analyses; *n* = 6 for ACC analyses; *P* < 0.05) assessing within and between treatments. (I) Photographs showing the root systems of plants grafted onto UD/WT, UD/*epi* and UD/*ACCD*, after 14 weeks growing under control and salt (75 mM NaCl) treatment. * indicates significant differences between control and salt treatment within each genotype according to the Mann–Whitney U test (*P* < 0.05). Results of two‐way ANOVA (*P* values reported) for genotype (G), treatment (T) and their interaction (G × T) are indicated in the top right of the panel. *, ** and *** indicate statistically significant differences at *P* < 0.05, *P* < 0.01 and *P* < 0.001, respectively.

To further evaluate the influence of altered ACC/ethylene status on root development, an additional experiment was conducted in non‐grafted seedlings grown under control and saline conditions. First, ethylene production measurements confirmed the contrasting ethylene condition, with *epi* producing the highest ethylene evolution, particularly under salinity, whereas *ACCD* plants consistently displayed the lowest values (Figure S1b). Second, salinity affected root system architecture (RSA) in a marked genotype‐dependent way (Figure S1), consistent with the genotype‐dependent RSA observed in adult grafted plants (Figure 1I). Quantitative WinRHIZO analyses revealed that *ACCD* seedlings developed significantly larger and more highly branched root systems than WT and *epi* plants, as evidenced by greater root length, root surface area, root volume and higher numbers of root tips and root forks under both control and saline conditions (Figure S1c–g). In contrast, *epi* plants generally exhibited the lowest values for most RSA parameters, particularly under salinity, while WT plants displayed intermediate responses. These differences in RSA traits were consistent with the contrasting ethylene status of the root genotypes and support the hypothesis that reduced ACC/ethylene eases adaptive RSA responses to salinity.

### Gas‐exchange parameters

Plants grafted onto *ACCD* and WT rootstocks registered the highest and lowest transpiration rate (*E*), stomatal conductance (*g*_*s*_) and net CO_2_ assimilation rate (*A*_*N*_) value under salinity, respectively (Table 1). Indeed, the WT grafted plants were the most negatively impacted in terms of *A*_*N*_, *E*, *g*_*s*_ and *Fv/Fm* with respect to control conditions, while the SPAD parameter, which reflects the chlorophyll content, was more affected by salinity in plants grafted onto *epi* rootstocks (Table 1). Overall, *ACCD*‐grafted plants maintained the highest values of E, *gs, A*_*N*_ and SPAD under salinity, whereas WT (*E, g*_*S*_, *A*_*N*_) and *epi* (SPAD) grafts suffered the greatest reductions in these parameters. Interestingly, *epi* grafts performed intermediate in gas‐exchange parameters between WT and *ACCD* ones in whatever conditions.

### Leaf and flower nutrient status

Salinity usually impairs plant fitness by altering nutrient status and inducing Na‐toxic effects in vegetative and reproductive organs. Under control conditions, plants grafted onto *ACCD* rootstocks had higher leaf nutrient (Ca, B, Fe, Zn) status than WT and *epi*, while WT grafts registered the highest Mg concentrations and *epi* the lowest (Table 2). Salinity decreased leaf nutrients in WT (N, P, S, Ca, Mg, B, Mn), *epi* (S, Ca, Mn) and *ACCD* (Mn) grafts in a genotype‐dependent way. Indeed, under salinity, *ACCD* grafts registered the highest macro (P, S, Ca) and micro (B, Fe, Mn, Cu) concentrations in the mature leaves compared with the other grafts (Table 2). However, *epi*‐grafted plants registered the lowest N, S, Ca, Mg, Mn and Cu and the highest Na concentrations in the salinised leaves. Similarly, under salinity conditions, the flowers of the *epi* grafts registered the lowest K, S, Ca, Mg, B, Fe, Mn, Cu and Zn and the highest Na concentrations, while WT registered the lowest Na and highest K/Na ratio in this organ. Interestingly, *ACCD* grafts maintained stable Ca concentration in both leaves and flowers under salinity, while an increase in the flowers in detriment of the leaves was observed in WT and *epi* grafts (Table 2).

**Table 2:** Macro‐ and micronutrients in flower and leaf

	Control			Salt			*P* (ANOVA)		
	UD/WT	*UD/epi*	*UD/ACCD*	UD/WT	*UD/epi*	*UD/ACCD*	G	T	G × T
*Flower nutrients* (*mgg−1DW*)									
C	427.36 ± 1.84^ab^	420.00 ± 4.27^b^	421.13 ± 3.89^b^	434.45 ± 2.49^a^	425.91 ± 0.44^ab^	421.99 ± 1.88^b^	0.015*	0.079	0.543
N	42.07 ± 1.15^ab^	41.23 ± 1.68^b^	40.72 ± 1.03^b^	45.25 ± 1.08^a^	40.71 ± 0.73^b^	40.46 ± 0.17^b^	0.041*	0.424	0.251
P	6.54 ± 0.14^ab^	6.86 ± 0.21^a^	7.06 ± 0.27^a^	5.84 ± 0.18^b^	6.33 ± 0.07^ab^	6.72 ± 0.36^a^	0.023	0.013*	0.738
K	24.71 ± 0.60^a^	26.93 ± 0.78^a^	24.76 ± 1.35^a^	26.35 ± 0.63^a^	21.39 ± 0.57^b^	24.61 ± 0.89^a^	0.320	0.078	0.003**
Na	0.39 ± 0.02^d^	0.51 ± 0.03^d^	0.62 ± 0.05^cd^	1.09 ± 0.28^bc^	1.94 ± 0.11^a^	1.26 ± 0.08^b^	0.020*	<0.001***	0.048*
K/Na	63.68 ± 2.15^a^	53.28 ± 5.33^ab^	42.29 ± 2.81^b^	28.95 ± 6.28^c^	11.15 ± 0.92^d^	18.74 ± 1.37^cd^	0.002**	<0.001***	0.119
S	3.50 ± 0.14^b^	3.94 ± 0.10^ab^	4.56 ± 0.13^a^	4.00 ± 0.26^ab^	3.41 ± 0.11^b^	4.31 ± 0.29^a^	0.003**	0.592	0.061
Ca	6.07 ± 0.62^b^	5.84 ± 0.33^b^	7.02 ± 0.42^ab^	7.48 ± 1.38^a^	5.49 ± 0.30^ab^	7.71 ± 0.73^a^	0.072	0.010*	0.450
Mg	3.05 ± 0.20^bc^	3.82 ± 0.08^a^	3.57 ± 0.09^ab^	3.37 ± 0.05^ab^	2.68 ± 0.01^c^	3.01 ± 0.12^bc^	0.321	0.182	<0.001***
B	0.04 ± 0.00^b^	0.06 ± 0.00^a^	0.05 ± 0.00^ab^	0.05 ± 0.00^ab^	0.05 ± 0.00^b^	0.05 ± 0.01^ab^	0.114	0.255	0.119
Fe	0.14 ± 0.04^a^	0.14 ± 0.02^a^	0.15 ± 0.03^a^	0.14 ± 0.01^a^	0.07 ± 0.01^a^	0.10 ± 0.01^a^	0.479	0.071	0.456
Mn	0.05 ± 0.00^bc^	0.06 ± 0.00^ab^	0.06 ± 0.00^a^	0.07 ± 0.00^a^	0.05 ± 0.00^c^	0.06 ± 0.01^ab^	0.187	0.289	<0.001***
Cu	0.01 ± 0.00^bc^	0.02 ± 0.00^ab^	0.02 ± 0.00^a^	0.014 ± 0.00^bc^	0.01 ± 0.00^c^	0.01 ± 0.00^bc^	0.204	0.017*	0.086
Zn	0.04 ± 0.00^bc^	0.05 ± 0.00^ab^	0.05 ± 0.01^a^	0.04 ± 0.00^bc^	0.04 ± 0.00^c^	0.04 ± 0.00^bc^	0.057	0.026*	0.024*
*Leaf nutrients* (*mgg* ^*−1*^ *DW*)									
C	385.40 ± 1.64^a^	387.68 ± 1.06^a^	379.87 ± 6.33^a^	389.51 ± 4.19^a^	377.11 ± 11.26^a^	386.97 ± 1.86^a^	0.644	0.965	0.272
N	36.16 ± 1.38^b^	37.29 ± 0.54^b^	34.65 ± 0.66^b^	41.00 ± 0.51^a^	34.19 ± 2.02^b^	34.62 ± 0.97^b^	0.008**	0.551	0.010*
P	5.05 ± 0.21^a^	5.46 ± 0.52^a^	5.35 ± 0.93^a^	2.78 ± 0.23^b^	4.52 ± 1.02^ab^	6.42 ± 0.40^a^	0.029*	0.207	0.072
K	20.10 ± 1.67^a^	20.88 ± 0.96^a^	20.07 ± 0.90^a^	20.73 ± 0.74^a^	23.04 ± 0.37^a^	21.32 ± 1.49^a^	0.362	0.167	0.792
Na	1.41 ± 0.07^c^	1.86 ± 0.14^c^	1.73 ± 0.20^c^	5.61 ± 1.05^b^	7.72 ± 0.74^a^	4.43 ± 0.70^b^	0.029*	<0.001***	0.055
K/Na	14.31 ± 1.12^a^	11.50 ± 1.25^a^	11.13 ± 0.86^a^	4.07 ± 0.71^b^	3.07 ± 0.30^b^	5.44 ± 1.43^b^	0.201	<0.001***	0.128
S	24.42 ± 0.85^a^	19.80 ± 3.10^ab^	20.96 ± 0.26^ab^	18.51 ± 0.80^b^	11.89 ± 1.19^c^	20.82 ± 0.43^ab^	0.002**	<0.001***	0.040*
Ca	47.00 ± 1.46^b^	42.19 ± 1.43^c^	52.33 ± 2.65^a^	35.55 ± 0.51^d^	32.28 ± 1.69^d^	49.17 ± 1.62^ab^	<0.001***	<0.001***	0.046*
Mg	5.30 ± 0.26^a^	4.02 ± 0.10^cd^	4.69 ± 0.03^b^	4.48 ± 0.15^bc^	3.69 ± 0.01^d^	4.23 ± 0.13^bc^	<0.001***	0.001**	0.310
B	0.10 ± 0.00^bc^	0.10 ± 0.01^b^	0.14 ± 0.01^a^	0.07 ± 0.00^c^	0.09 ± 0.01^bc^	0.11 ± 0.00^b^	0.002**	0.010*	0.688
Fe	0.16 ± 0.01^ab^	0.15 ± 0.01^b^	0.18 ± 0.00^a^	0.16 ± 0.01^ab^	0.16 ± 0.00^ab^	0.18 ± 0.01^a^	0.015*	0.550	0.777
Mn	0.14 ± 0.01^a^	0.12 ± 0.00^b^	0.12 ± 0.00^b^	0.09 ± 0.00^c^	0.07 ± 0.00^d^	0.10 ± 0.00^c^	<0.001***	<0.001***	0.006**
Cu	0.01 ± 0.00^c^	0.01 ± 0.00^ab^	0.01 ± 0.00^bc^	0.01 ± 0.00^ab^	0.01 ± 0.00^bc^	0.01 ± 0.00^a^	0.196	0.025*	0.002**
Zn	0.01 ± 0.00^b^	0.01 ± 0.00^ab^	0.02 ± 0.00^a^	0.02 ± 0.00^a^	0.01 ± 0.00^ab^	0.02 ± 0.00^a^	0.191	0.084	0.096

Total carbon (C), total nitrogen (N), phosphorus (P), potassium (K), sodium (Na), K/Na ratio, sulphur (S), calcium (Ca), magnesium (Mg), boron (B), iron (Fe), manganese (Mn), copper (Cu) and zinc (Zn) concentrations in flowers and leaves of the tomato cv UniDarkwin (UD) grafted onto MT (UD/WT), *epi* (high ethylene production, UD/*epi*) and *ACCD* (low ethylene production, UD/*ACCD*) rootstocks after 14 weeks growing under control and salt (75 mM NaCl) treatment. Values indicate mean ± SE. Different letters indicate statistically significant differences among graft combinations (*n* = 6; *P* < 0.05) within and between treatments. Results of two‐way ANOVA (*P* values reported) for genotype (G), treatment (T) and their interaction (G × T) are indicated on the right side of the Table. *, ** and *** indicate statistically significant difference at *P* < 0.05, *P* < 0.01 and *P* < 0.001, respectively.

### Hormonal profiling

Ethylene action is often associated with crosstalk with other hormones involved in plant development and stress responses, such as abscisic (ABA), jasmonic (JA) and salicylic (SA) acids, gibberellins (GAs), cytokinins (CKs) and auxins (Chen et al., 2021). For a better understanding of rootstock‐mediated ACC/ethylene production on local and systemic effects of the plants, a comprehensive analysis of main plant hormones and some ABA derivatives was performed in root, leaf and flower tissues.

### 1‐aminocyclopropane‐1‐carboxylic and abscisic acids

Under control conditions, the ethylene‐precursor ACC was undetectable in leaves and detected at low levels in flowers across all graft combinations. However, root ACC values in *epi*‐grafted plants were 3‐times higher than in WT and *ACCD* ones in the absence of stress (Figures 1 and 2; Table S2), irrespective of genotype; salinity increased ACC levels in roots, leaf xylem sap, leaves and flowers. Notably, plants grafted onto *epi* rootstocks displayed a 3.5 and 110‐fold rise in ACC in the roots, compared to WT and *ACCD*‐grafted plants, respectively (Figure 1D) and registered a twofold increase in leaves and flowers compared to the other grafts (Figure 1A,B). Similarly, plants grafted onto *epi* rootstocks displayed 1.24‐ and 1.62‐fold higher ACC concentrations in leaf xylem sap compared with WT‐ and *ACCD*‐grafted plants, respectively (Figure 1C). Those results are consistent with the higher ACC and ethylene production observed in young seedling *epi* roots and shoots in the absence of any imposed stress, when compared to the wild‐type VFN8 (Fujino et al., 1988) and with the comparative analysis in seedlings of the three genotypes under control and saline conditions (Figure S1b). Indeed, *ACCD* rootstocks consistently registered the lowest ACC concentrations across all organs and conditions, especially in roots, which showed no ACC increase under salinity. Overall, *ACCD* rootstocks decreased ACC evolution in both roots and the aerial part of the plant, particularly in the flowers, potentially influencing ethylene‐dependent local (roots) and systemic (leaves and fruits) growth responses.

**Figure 2:**
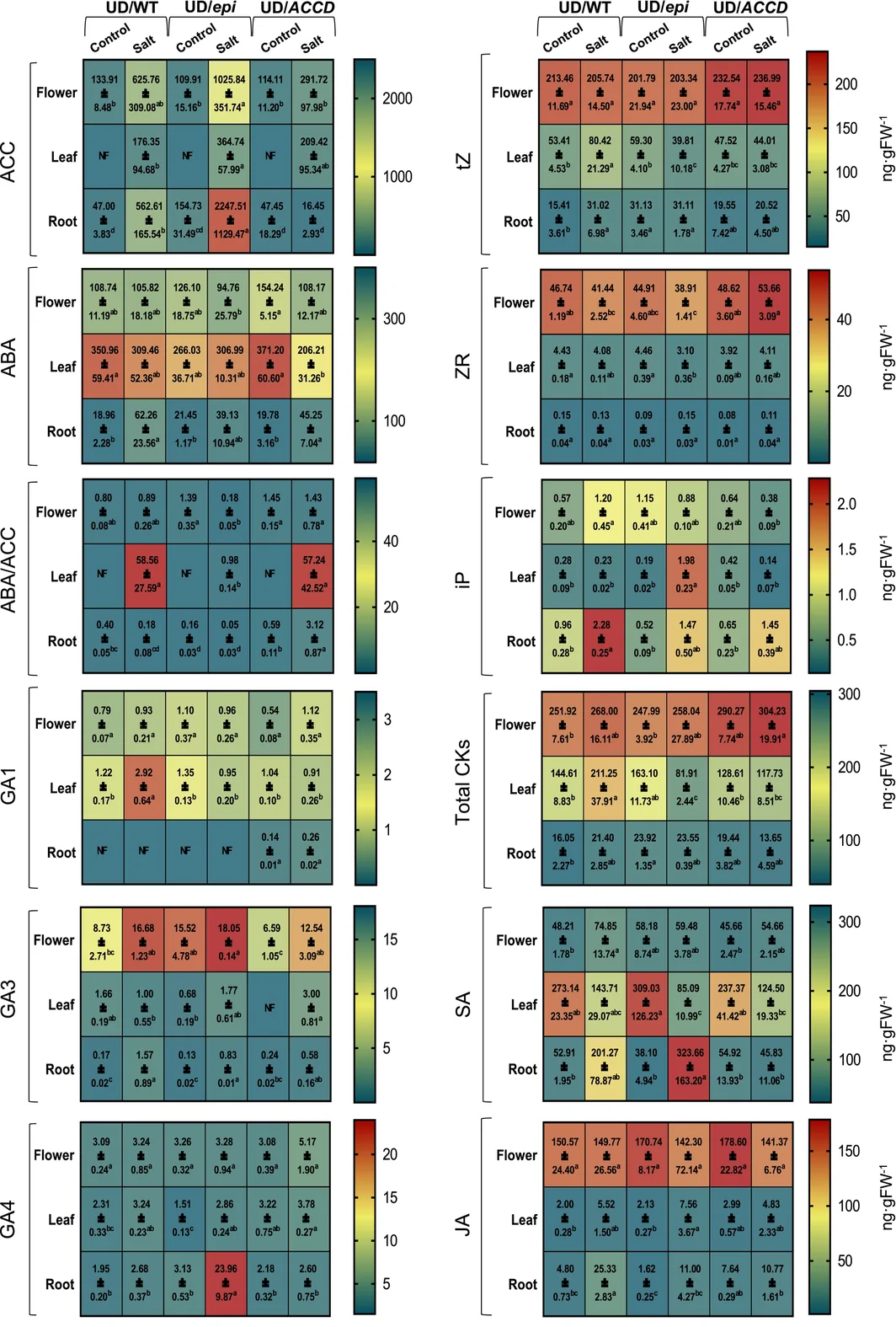
Heatmap of plant hormone concentrations: 1‐aminocyclopropane‐1‐carboxylic acid (ACC) and abscisic acid (ABA) and ABA/ACC ratio, gibberellins GA1, GA3 and GA4, trans‐zeatin (tZ), zeatin riboside (ZR), isopentenyl adenine (iP), total cytokinins (CKs) (tZ + ZR + iP), jasmonic acid (JA) and salicylic acid (SA) concentrations in flowers, leaves, and roots of the tomato cv UniDarkwin (UD) grafted onto MT (UD/WT), *epi* (high ethylene production, UD/*epi*) and *ACCD* (low ethylene production, UD/*ACCD*) rootstocks after 14 weeks growing under control and salt (75 mM NaCl) treatment. Hormone concentrations are presented as ng/g of fresh tissue, with colour gradients ranging from green (lowest concentrations) to red (highest concentrations). Different letters indicate significant differences among graft combinations (*n* = 6; *P* < 0.05) within and between treatments.

Genotype and treatment mediated alterations in ACC metabolism and levels were further supported by changes in its precursor S‐adenosyl‐L‐methionine (SAM) and glutamyl (GACC), malonyl (MACC) and jasmonic (JA‐ACC) ACC conjugates (Figure S2). Under control conditions, *ACCD* grafts accumulated higher SAM levels in roots, which may reflect a reduced conversion of SAM to ACC. Likewise, contrasting GACC, JA‐ACC and MACC levels in *ACCD* and *epi* roots and leaves were consistent with the altered ACC pools observed in these grafts, being intermediate in WT ones. The elevated GACC concentrations in *epi* leaves under both conditions suggest an increased apoplastic transport of ACC through the xylem (Vanderstraeten & Van Der Straeten, 2017). No significant differences were found between graft combinations in root, leaf and flower ABA concentrations under control conditions. Salinity increased root ABA levels by 2–3 times, but only significantly in WT and *ACCD* rootstocks, while the stress caused a significant decrease in leaf ABA concentration in *ACCD* grafts, compared to control conditions (Figure 2; Table 3, Table S2). The roots of *ACCD* grafts registered the highest ABA/ACC ratio under salinity compared to the other grafts' combinations, while *epi* rootstocks registered the lowest values (Figure 2; Table S2).

**Table 3:** Concentrations of metabolites related to ABA and biosynthesis, catabolism and conjugation of ABA in flowers, leaves and roots

			Control			Salt			*P* (ANOVA)		
			UD/WT	*UD/epi*	*UD/ACCD*	UD/WT	*UD/epi*	*UD/ACCD*	G	T	G × T
Flower (ng·gFW^−1^)	ABA		108.74 ± 11.19^ab^	126.10 ± 18.75^ab^	154.24 ± 5.15^a^	105.82 ± 18.18^ab^	94.76 ± 25.79^b^	108.17 ± 12.01^ab^	0.256	0.047*	0.356
	ABA biosynthesis	ABA aldehyde	NF	NF	NF	0.0087 ± 0.0017	NF	NF	ND	ND	ND
	ABA catabolism	PA	0.5560 ± 0.0404^c^	0.6958 ± 0.0175^ab^	0.7459 ± 0.0155^a^	0.5525 ± 0.0194^c^	0.5996 ± 0.0396^bc^	0.5665 ± 0.0487^c^	0.010*	0.003**	0.047*
		DPA	0.5817 ± 0.0423^b^	0.6229 ± 0.0477^b^	0.4832 ± 0.0289^b^	0.6621 ± 0.1007^b^	1.2390 ± 0.2172^a^	0.5897 ± 0.0871^b^	0.001**	0.002**	0.015*
		DPAG	22.9243 ± 3.0530^a^	20.0256 ± 2.8654^a^	23.3147 ± 1.3615^a^	23.7181 ± 3.4685^a^	18.5926 ± 2.6782^a^	22.7018 ± 4.5634^a^	0.430	0.877	0.941
		neoPA	0.3765 ± 0.0320^b^	0.5091 ± 0.0189^ab^	0.4831 ± 0.0341^ab^	0.3568 ± 0.1280^b^	0.6234 ± 0.0469^a^	0.4613 ± 0.0414^ab^	0.029*	0.666	0.556
	ABA conjugate	ABA‐GE	0.0388 ± 0.0026^c^	0.0577 ± 0.0162^bc^	0.0141 ± 0.0042^c^	0.1579 ± 0.0430^a^	0.1394 ± 0.0025^a^	0.1036 ± 0.0249^ab^	0.141	<0.001***	0.650
Leaf (ng·gFW^−1^)	ABA		350.96 ± 59.41^a^	266.03 ± 36.71^ab^	371.20 ± 60.60^a^	309.46 ± 52.36^ab^	306.99 ± 10.31^ab^	206.21 ± 31.26^b^	0.606	0.193	0.157
	ABA biosynthesis	ABA aldehyde	0.1097 ± 0.0175^ab^	0.1230 ± 0.0029^ab^	0.2364 ± 0.0103^a^	0.2171 ± 0.0140^ab^	0.2388 ± 0.1022^a^	0.0825 ± 0.0338^b^	0.910	0.548	0.014*
	ABA catabolism	PA	4.9441 ± 0.6056^a^	3.9166 ± 0.1513^ab^	4.0400 ± 0.5562^ab^	3.2222 ± 0.3449^b^	3.8740 ± 0.1708^ab^	3.1680 ± 0.6402^b^	0.386	<0.001***	0.345
		DPA	0.7061 ± 0.0500^b^	0.9802 ± 0.0892^b^	0.8471 ± 0.0667^b^	0.9552 ± 0.0517^b^	2.5734 ± 1.1145^a^	1.3069 ± 0.1204^ab^	0.161	0.070	0.350
		DPAG	6.0068 ± 0.5069^b^	5.2631 ± 0.4437^b^	5.8391 ± 0.9469^b^	13.0042 ± 1.0725^a^	12.3005 ± 1.8144^a^	11.1623 ± 1.8990^a^	0.745	<0.001***	0.762
		neoPA	0.4664 ± 0.1291^a^	0.3539 ± 0.0915^a^	0.2391 ± 0.0725^ab^	0.0329 ± 0.0057^b^	0.0180 ± 0.0047^b^	0.0391 ± 0.0112^b^	0.388	<0.001***	0.354
	ABA conjugate	ABA‐GE	0.7208 ± 0.0196^c^	0.7575 ± 0.1373^bc^	0.6558 ± 0.0860^c^	1.3741 ± 0.3496^bc^	2.4916 ± 0.5583^a^	1.5834 ± 0.1873^b^	0.097	<0.001***	0.148
Root (ng·gFW^−1^)	ABA		18.96 ± 2.28^b^	21.45 ± 1.17^b^	19.78 ± 3.16^b^	62.26 ± 23.56^a^	39.13 ± 10.94^ab^	45.25 ± 7.04^ab^	0.633	0.006**	0.520
	ABA biosynthesis	ABA aldehyde	0.0042 ± 0.0003^b^	0.0071 ± 0.0018^b^	0.0236 ± 0.0108^b^	0.4239 ± 0.1091^b^	1.3165 ± 0.5411^a^	0.3839 ± 0.0010^b^	0.179	0.006**	0.172
	ABA catabolism	PA	0.0046 ± 0.0004^b^	NF	NF	0.2143 ± 0.1475^a^	0.2755 ± 0.1552^a^	0.0111 ± 0.0009^b^	ND	ND	ND
		DPA	0.3092 ± 0.0534^b^	0.3758 ± 0.0152^ab^	0.3551 ± 0.0698^ab^	0.5756 ± 0.1208^ab^	1.1354 ± 0.5056^a^	0.8985 ± 0.2761^ab^	0.444	0.016*	0.596
		DPAG	23.8627 ± 0.8660^b^	22.8336 ± 1.5394^b^	36.1180 ± 5.4528^a^	30.1420 ± 5.2315^ab^	28.8542 ± 3.5640^ab^	37.3092 ± 1.6277^a^	0.021*	0.157	0.758
		neoPA	NF	NF	NF	0.0136 ± 0.0057^a^	0.0111 ± 0.0057^a^	0.0147 ± 0.0045^a^	ND	ND	ND
	ABA conjugate	ABA‐GE	0.0038 ± 0.0004^b^	NF	0.0050 ± 30.004^b^	0.0074 ± 0.0008^ab^	0.0229 ± 0.0102^a^	0.0041 ± 0.0005^b^	ND	ND	ND

Concentrations of metabolites related to ABA biosynthesis (ABA aldehyde), ABA catabolism (phaseic acid, PA; dihydrophaseic acid, DPA; dihydrophaseic acid glucoside DPAG; neophaseic acid, neoPA) and ABA conjugates (ABA glucosyl ester, ABA‐GE) in flowers, leaves and roots of the tomato cv UniDarkwin (UD) grafted onto MT (UD/WT), *epi* (high ethylene production, UD/*epi*) and *ACCD* (low ethylene production, UD/*ACCD*) rootstocks after 14 weeks growing under control and salt (75 mM NaCl) treatment. Values indicate mean ± SE. Different letters indicate statistically significant differences among graft combinations (*n* = 6; *P* < 0.05) within and between treatments. Results of two‐way ANOVA (*P* values reported) for genotype (G), treatment (T) and their interaction (G × T) are indicated in the right of the Table. *, ** and *** indicate statistically significant difference at *P* < 0.05, *P* < 0.01 and *P* < 0.001, respectively.

### ABA derivatives

Given the reported ethylene–ABA crosstalk in roots and its putative impact on the adaptation to salinity (Martínez‐Andújar et al., 2020, 2021), and to further explore the basis of ABA changes in *ACCD* grafts, the main ABA precursors, catabolites and conjugates were analysed. The immediate ABA precursor ABA aldehyde acts as the checkpoint for quick modulation of the hormone level in response to the environment (Xiong & Zhu, 2003). This precursor was twofold higher in the leaves of plants grafted onto *ACCD* rootstocks compared to WT and *epi* ones under control conditions, decreasing by 50% under salinity relative to control conditions, while the opposite was found in WT and *epi* rootstocks. This genotypic effect was not observed in flowers, where this ABA precursor was detected only in the WT grafted plants under salinity (Table 3). These results are consistent with those obtained for leaf ABA concentrations, since a reduction was observed in *ACCD* grafts under salinity (Figure 2; Table 3).

Genotypic differences were also observed in some metabolites related to ABA catabolism and conjugation, although the effects of salinity were generally more pronounced than those of rootstock genotype. Regarding the main ABA catabolic pathway through C‐8′ hydroxylation, no genotypic differences in PA concentration were observed in leaves under control conditions, while it dropped in plants grafted onto WT and *ACCD* rootstocks under salinity, remaining constant in *epi* ones. In contrast, DPA and its glycosylated form (DPAG) accumulated across all genotypes under salinity conditions. DPAG consistently exhibited strong increases in the three rootstocks, whereas the increase in DPA was less pronounced in plants grafted onto WT compared to those grafted onto *epi* and *ACCD*. Finally, leaf neoPA decreased with salinity in all genotypes, but to a lower extent in ACCD grafts. With respect to ABA conjugation, levels of ABA‐GE were similar in the leaves of all three graft combinations under control conditions. However, salinity increased leaf ABA‐GE concentrations in all graft combinations, ranging from 1.9‐fold in WT to 3.3‐fold in epi‐grafted plants, with the latter showing the highest ABA‐GE concentration. In flowers, no genotypic differences were observed for the analysed ABA‐related metabolites. Salinity decreased PA in *ACCD* grafts and increased ABA‐GE in all genotypes, but to a higher extent in WT and *epi* ones (Table 3). Overall, the results suggest that ACC/ethylene production in roots may contribute to the regulation of ABA metabolism in shoots through a putative root‐to‐shoot mediated mechanism.

### Gibberellins

Under control conditions, leaf GA1 concentrations were similar among graft combinations, GA3 was undetected in *ACCD*‐grafted plants, and the highest GA4 concentrations were recorded in *ACCD* rootstocks. Salinity increased gibberellin levels in the leaves in a genotype‐dependent way: GA1 in plants grafted onto WT rootstocks, GA3 in *ACCD* rootstocks and GA4 in the three grafts. No effect of genotype or treatment was observed in the flowers GA1 and GA4 concentrations, while the flower GA3 levels increased in response to salinity across all graft combinations. In roots, GA1 was detected only in *ACCD* grafts irrespective of the treatment, while GA3 increased under salinity in all graft combinations, but mainly in WT and *epi* rootstocks. Additionally, GA4 was specifically salt‐induced in the roots of *epi* grafts (Figure 2; Table S2).

### Cytokinins

Under control conditions, leaf tZ, ZR and iP did not differ among grafts. Salinity increased leaf tZ and iP in WT and *epi* grafts, respectively, while it decreased ZR in *epi* grafts. Indeed, total bioactive CKs (tZ + ZR + iP) increased under salinity in WT rootstocks, decreased in *epi* and remained stable in *ACCD* ones, relative to control conditions. In the flowers, no genotypic differences in CKs levels were observed under control conditions, while *ACCD* grafts registered the highest and lowest ZR and iP concentrations under salinity, respectively. In roots, ZR levels remain stable across all genotypes and treatments. Root tZ concentrations were not affected by salinity in *epi* and *ACCD* grafts, while they increased in WT rootstocks. No genotypic differences in root iP levels were detected under control conditions, but were induced under salinity, especially in WT grafts (Figure 2; Table S2).

### Salicylic and jasmonic acids

SA concentrations were 3–6 times higher in leaves than in flowers and roots, but no genotypic differences were found under control conditions. Salinity decreased SA in leaves and increased it in roots of *epi* grafts, while increasing flower SA in WT grafted plants (Figure 2; Table S2). Contrary to SA, JA concentration was 10–20 times higher in flowers than in leaves and roots, according to its predominant role in reproductive structures (Nie et al., 2025). Salinity increased JA in leaves and roots of *epi* and WT grafts, while this hormone remained unaltered in all organs of *ACCD* plants (Figure 2; Table S2).

### Flavonoids

Interestingly, the ethylene‐related rootstock genotype influenced the flavonoid contents in an organ and treatment dependent way. In roots, all the analysed flavonoids were detected, except for genistein (Table S3). Cyanidin concentrations were highly heterogeneous and increased under salinity in WT and *epi* grafts, while in *ACCD* grafts, they remained elevated in both treatments. Similarly, luteolin remained at higher levels while quercetin decreased in *ACCD* roots under saline treatment. Finally, taxifolin concentration strongly dropped in WT and *ACCD* roots under salt stress while it remained stable in *epi* grafts.

In leaves, quercetin was the only undetected flavonoid across genotypes and treatments (Table S3). Cyanidin increased in the three grafts under salinity. Genistein was only detected in WT under salinity. Luteolin decreased with salinity in all genotypes, especially in WT grafts. Taxifolin decreased under salinity in WT and *ACCD* grafts but increased in *epi* grafts plants, which accumulated twofold more taxifolin than other grafts.

In flowers, all the analysed flavonoids were found in a genotype and treatment dependent way (Table S3). Cyanidin increased under salinity in flowers of WT and *epi* grafts, while it remained stable in *ACCD* ones. Conversely, genistein increased under saline stress especially in WT and *ACCD* grafts, which registered 3.5 and 4.5 times more than *epi*, respectively. Contrarily, taxifolin increased in flowers of all rootstocks but mostly in *epi* grafts, where this compound accumulated until 2fold more than in the other rootstocks. Luteolin and quercetin were only detected in flowers of *ACCD* grafts.

Overall, *ACCD* grafts accumulated luteolin in roots in response to salinity, while taxifolin decreased. In leaves and flowers, taxifolin was more abundant in *epi* grafts under salinity, whereas genistein concentrations peaked in the flowers of plants grafted onto *ACCD* rootstocks under saline conditions.

### Amino acids content in flowers

Along with sugars, amino acids are largely transported to sink organs and are indicators of C/N balance and smooth source–sink relations. In flowers, amino acids are essential for pollen development, adaptation to stress and interaction with pollinators (Göttlinger & Lohaus, 2023). Under salinity, flowers of plants grafted onto the low ethylene rootstocks (*ACCD*) accumulated Ile, Pro and Arg in response to salinity and exhibited significantly higher levels of several amino acids, including Tyr, Ile, Pro, Hyp, Trp and Lys, compared to the other rootstocks (Table S4).

### Carbohydrates

Under saline conditions, *ACCD* grafts exhibited higher hexose (leaves and flowers) and sucrose (flowers) concentrations than WT and *epi* grafts, whereas salinity reduced these carbohydrates only in WT and *epi* rootstocks, compared to control conditions (Figure 3A; Table S2). In contrast, salinity significantly reduced leaf starch concentration only in WT grafts, while the highest concentrations were found in *epi* grafts under salinity (Figure 3A; Table S2). The differences in leaf starch contents were microscopically confirmed (Figure 3B).

**Figure 3:**
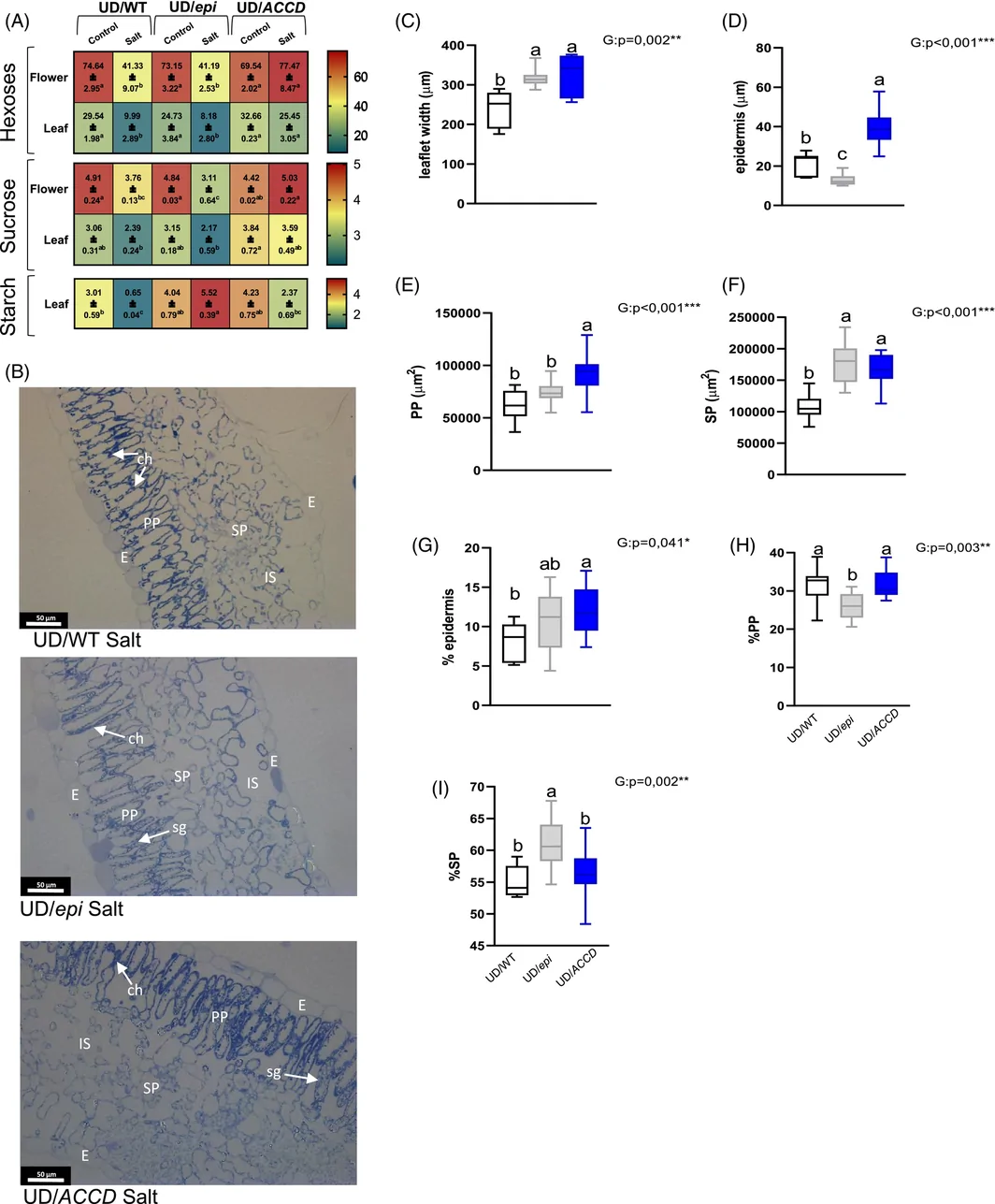
Carbohydrate allocation and leaf anatomical responses to salt stress in grafted tomato plants. (A) Heatmap of hexoses (fructose + glucose) and sucrose concentrations in flowers and leaves (μg·g^−1^ FW), as well as starch levels in leaves of the tomato cv. UniDarkwin grafted onto MT (UD/WT), *epi* (high ethylene production, UD/*epi*) and *ACCD* (low ethylene production, UD/*ACCD*) rootstocks after 14 weeks growing under control and salt (75 mM NaCl) treatment. Sucrose and hexoses concentrations are presented as μg.g^−1^, and starch is presented as μg.mg^−1^ of fresh tissue, with colour gradients ranging from green (lowest concentrations) to red (highest concentrations). (B) Optical microscopy images showing longitudinal sections of leaf anatomy of the commercial tomato cv. UniDarkwin plants grafted onto WT, *epi* and *ACCD* rootstocks after 13 weeks of salt treatment (75 mM NaCl), showing the different leaf structures, including the epidermis (E), palisade parenchyma (PP), spongy parenchyma (SP), intercellular space (IS), as well as chlorophyll (ch) and starch granules (sg). (C–I) Leaf morphometric traits of the same leaves, including leaf (C) and epidermis (D) thickness, areas of PP (E) and SP (F), and percentages of epidermis (G), PP (H) and SP (I) relative to the total area, including the IS. Bars indicate mean values ± SE. Different letters indicate significant differences among graft combinations (*n* = 6 for carbohydrate analyses; *n* = 3 for anatomical traits, based on six sections per plant; *P* < 0.05).

### Leaf anatomy

Changes in leaf morphological structure are an important adaptive response to salinity stress (Acosta‐Motos et al., 2017). Indeed, although a constitutive genotypic effect cannot be ruled out, leaf anatomy revealed some rootstock‐dependent differences under salinity (Figure 3B–I). Leaves from plants grafted onto WT rootstocks were thinner than in *epi* and *ACCD* grafts (Figure 3C), while the leaf epidermis of *ACCD* grafts was thicker compared to that from WT and *epi* rootstocks (Figure 3D,G). The percentage of palisade parenchyma (PP) relative to the total leaf area in *epi* grafts was 17.5% lower than in other graft combinations (Figure 3E,H). Additionally, the spongy parenchyma (SP) in the leaf of plants grafted onto *epi* rootstocks appeared more disorganised compared to the other rootstock genotypes (Figure 3B,F,I).

### Leaf transcriptomic analysis

To characterise the systemic responses mediated by the ethylene‐related rootstock genotypes, RNA‐seq analyses were performed in leaves. RNA‐seq showed that salinity was the main source of transcriptomic variation (Figure S3a). Over 79% of the RNA‐Seq was mapped to the SL4 genome, observing a total of 20 808 transcripts after normalisation under salinity. Each cluster comprised over 1400 genes that exhibited upregulation exclusively within each genotype between treatments (Figure S3b).

Gene Ontology (GO) enrichment analysis indicated that these clusters were mainly associated with biological processes (BP), such as redox metabolism and response to abiotic stress, among others. The blue cluster showed an enrichment in GO BP terms related to photosynthesis, carotenoids and chloroplast organisation (Figure S3c). The violet cluster exhibited enrichment in terms linked to protein folding, response to oxidative stresses and RNA modification (Figure S3d). Finally, the green cluster enriched terms were related to sterol and steroid biosynthetic and metabolic processes (Figure S3e,f; Table S15).

Differential expression analysis identified 1385 differentially expressed genes (DEGs) across genotypes and treatments, with most associated with genotypic responses to salinity, as shown in the C7‐C9 contrasts (Figure 4A). Notably, *ACCD* grafts (C9 contrast) exhibited the highest number of deregulated genes, while WT rootstocks (C7 contrast) had the fewest DEGs (Figure 4A–C). GO enrichment highlighted stress‐related processes across all genotypes, with distinct differences among them (Figure 4D–F). Specifically, *epi* grafts (C8) showed enrichment in carbohydrate metabolic processes and transmembrane transport (Figure 4E). In contrast, *ACCD* grafts (C9) harboured numerous BP genes associated with both carbohydrate and sterol metabolic processes (Figure 4F; Table S15). With regard to sterol metabolism, 8 tomato orthologues from *A. thaliana* exhibited higher expression in the leaf of *ACCD* rootstocks under salt treatment (Figure 4I, Figure S3f). The *ACCD* versus WT comparison (C4) highlighted four tomato phosphatases orthologues of *Arabidopsis* genes (*SPX3, PAP17* and *PEPC1*/*PS2*, respectively) associated with phosphate starvation (Figure 4H; Table S15). Additionally, *ACCD* grafts under salinity (C9) exhibited upregulation of three *cytochrome P450* genes (Solyc06g074420, Solyc01g109150, Solyc05g021390) (Tables S5, S11 and S12), along with hormone‐related genes, including those involved in ethylene signalling *ERF* (Solyc04g051360) (Tables S5, S6 and S12).

**Figure 4:**
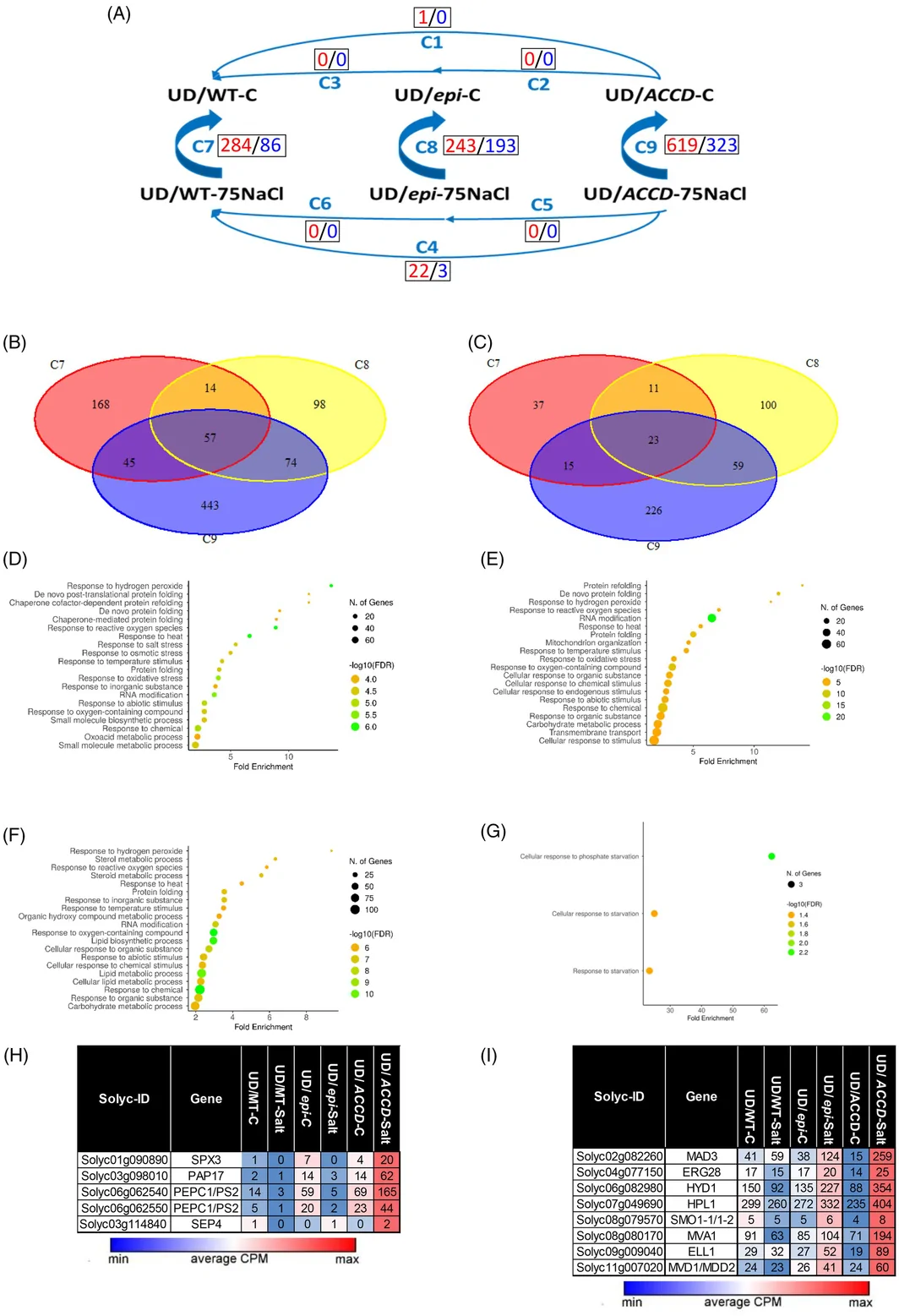
Differential expression and GO enrichment analysis highlight key molecular pathways modulated under salt stress. (A) Schematic representation of the different contrasts (C1‐C9) used for differential expression analysis together with the number of differentially expressed genes (DEGs) identified in each contrast. The numbers shown next to each contrast indicate the number of upregulated (red) and downregulated (blue) DEGs. Contrasts showing 0 did not contain any DEGs. Venn diagram showing the intersection of upregulated (B) and downregulated (C) DEGs identified in the contrasts C7, C8 and C9. GO biological process enrichment analysis of DEGs clusters in contrasts C7 (D), C8 (E), C9 (F) and C4 (G). Expression profiles of tomato orthologues of *A. thaliana* found in C4 contrast (H, I). Expression values are represented as average counts per million (CPMs).

Additional transcriptomic analyses revealed differential regulation of genes associated with phosphate metabolism, sodium homeostasis and sterol biosynthesis among graft combinations under salinity (Figure S4; Table S15). In particular, *ACCD* grafts showed enhanced expression of several sterol biosynthesis‐related genes, together with distinct expression patterns in phosphate‐ and sodium‐related pathways, supporting improved membrane and ionic homeostasis under saline conditions.

To validate RNA‐seq analysis, the expression of five representative genes (*ACO1, ACO2*, *CYP707A1, TAS14 and PIP1.2*) was analysed by qRT‐PCR. These genes are involved in different biological processes, including ethylene biosynthesis (*ACO1*, *ACO2*), ABA catabolism (*CYP707A1*), stress responses (*TAS14*) and aquaporin function (*PIP1.2*), which were confirmed through qRT‐PCR analysis (del Mar Parra et al., 1996; Reuscher et al., 2013). qRT‐PCR validation revealed an expression pattern consistent with those identified through RNA‐seq, supporting the reliability of RNA‐seq analysis (Figure S5).

### Expression of ACC‐to‐ethylene forming genes in roots and leaves (qRT‐PCR)

To further support the higher ethylene production in *epi* grafts, as suggested by the increased ACC levels (Figures 1B,D and 2), the expression of two *ACO* genes, *ACO1* and *ACO2*, responsible for converting ACC into ethylene, was measured in both roots and leaves (Figure 1B). In roots, both genes showed a clear genotype‐dependent pattern, which was further modulated by salinity. Specifically, in *epi* rootstocks, these genes were significantly upregulated under both control and saline conditions, consistent with the increased ACC levels and enhanced ethylene biosynthesis in different organs of this genotype (Fujino et al., 1988). In contrast, *ACO1* and *ACO2* expression was reduced in *ACCD* roots under salinity, compared to others (Figure 1G,H), aligning with the lower ACC concentrations and diminished ethylene production in these rootstocks (Figure 1D). In leaves, *ACO1* expression was influenced by salinity, although the effect was not statistically significant, while *ACO2* expression was influenced by both, genotype and treatment significantly (Figure 1E,F, Figure S5; Table 4, Table S6).

**Table 4:** Hormone‐related genes (ethylene, ABA, GA, CK and JA) differentially expressed in leaves

Solyc‐ID	Gene	UD/MT‐C	UD/MT‐Salt	UD/*epi‐C*	UD/*epi*‐Salt	UD/*ACCD*‐C	UD/*ACCD*‐Salt	
Solyc07g049530	*ACO1/EFE*	76.32	200.41	122.51	152.99	109.48	225.72	Ethylene
Solyc12g005940	*ACO2/EFE*	0.28	0.50	0.84	1.98	0.60	1.08	
Solyc08g079750	*ACS12*	70.29	67.85	82.71	89.92	95.12	80.00	
Solyc03g007070	*ACS10*	31.26	32.45	30.92	43.66	36.44	39.59	
Solyc08g016720	*NCED3*	10.37	5.55	14.94	5.50	21.69	8.52	ABA
Solyc07g056570	*NCED3*	32.16	34.82	32.76	23.60	45.83	26.69	
Solyc10g079860	*BG1*	67.07	98.49	155.37	31.63	82.07	121.47	
Solyc08g005610	*CYP707A1*	0.28	0.80	0.45	1.30	0.23	3.57	
Solyc04g078900	*CYP707A3*	22.68	12.90	22.19	44.59	11.40	39.93	
Solyc03g116890	*WRKY40*	3.15	7.66	3.31	7.20	3.09	11.10	
Solyc09g015380	*PYL5/RCAR1*	0.52	0.56	0.65	0.21	0.40	1.24	
Solyc05g056550	*SNRK2.6/OST1*	0.41	0.39	0.58	0.63	0.30	0.51	
Solyc02g090390	*SNRK2.6/OST1*	7.95	6.97	9.83	10.96	8.70	14.26	
Solyc03g096670	*PP2C*	18.16	46.55	25.50	49.16	34.68	37.36	
Solyc07g061730	*GA2OX3*	0.03	0.17	0.01	0.19	0.01	0.51	GA
Solyc09g074270	*OsGID1*	7.82	19.72	19.55	78.64	13.48	38.04	
Solyc06g008870	*GID1B*	12.76	22.90	22.60	24.11	20.84	28.53	
Solyc11g010850	*CLA1/DXS1*	44.50	80.77	43.70	126.38	15.34	241.27	
Solyc01g067890	*CLA1/DXS1*	66.17	80.91	58.08	84.13	59.63	107.23	
Solyc08g066950	*CLA1/DXS1*	17.50	16.47	19.21	20.80	16.94	23.33	
Solyc01g093980	*GA20ox2*	0.25	0.04	0.08	0.05	0.04	0.51	
Solyc06g066820	*GA3ox2*	0.24	0.10	0.20	0.82	0.11	0.61	
Solyc04g081290	*LOG3*	52.04	105.34	72.61	182.47	79.29	110.66	CK
Solyc08g062820	*LOG5*	6.60	12.39	8.77	20.32	7.35	18.21	
Solyc11g066960	*IPT5*	4.18	2.87	4.19	3.82	3.30	4.56	
Solyc04g016190	*ZOGL4*	0.22	0.21	0.24	0.74	0.09	1.14	
Solyc10g079350	*ZOGL1*	15.37	68.87	11.48	23.41	6.32	35.62	
Solyc11g066670	*ZOG6*	226.39	317.76	183.37	453.62	112.34	621.68	
Solyc04g016200	*ZOG3*	6.50	8.33	7.97	14.67	5.23	22.05	
Solyc05g053120	*ZOGL5*	19.00	13.36	16.77	17.07	8.29	34.69	
Solyc11g066680	*ZOG7*	43.58	50.98	39.57	60.45	25.49	81.75	
Solyc12g009940	*ZOGL3*	2.28	4.26	2.94	4.62	2.63	6.09	
Solyc06g062290	*ZOG4*	0.41	1.01	0.64	1.04	0.51	1.14	
Solyc12g009930	*ZOG5*	227.41	91.93	207.53	169.26	169.47	285.79	
Solyc01g098400	*AHPT1L*	0.33	0.67	0.48	0.96	0.48	2.59	
Solyc06g061030	*ARR‐B12*	45.86	91.24	69.96	108.36	63.16	138.69	
Solyc05g014260	*ARR‐B5*	46.89	34.00	46.63	71.53	45.13	88.14	
Solyc08g077230	*ARR‐B11*	45.90	52.65	40.19	179.25	47.45	75.67	
Solyc08g081960	*CRF2*	0.83	0.67	0.95	0.77	0.72	1.25	
Solyc03g007460	*CRF4*	0.21	0.45	0.58	0.39	0.55	0.72	
Solyc06g051840	*CRF6*	0.39	0.24	0.28	0.66	0.29	0.38	
Solyc08g014000	*LOX6*	2.24	13.36	2.53	9.00	1.27	63.21	JA
Solyc03g122340	*LOX2*	71.79	250.12	75.50	184.48	67.49	311.78	
Solyc07g042170	*JAZ1*	40.09	65.06	30.13	79.64	24.14	92.42	
Solyc02g085730	*AOC1/2/3/4*	164.81	310.37	187.94	281.94	131.16	383.68	
Solyc12g009220	*JAZ2*	4.54	31.11	6.67	8.77	5.41	20.31	
Solyc07g054580	*JAR1*	3.99	5.09	5.46	1.79	1.66	4.73	

Differentially expressed hormone‐related genes (ethylene, ABA, GA, CK and JA) in leaves of the tomato cv UniDarkwin (UD) grafted onto MT (UD/WT), *epi* (high ethylene production, UD/*epi*) and *ACCD* (low ethylene production, UD/*ACCD*) rootstocks after 14 weeks growing under control and salt (75 mM NaCl) treatment. Expression values are expressed as average counts per million (CPMs). A colour gradient represents the expression level, with deep red indicating the highest values and deep blue indicating the lowest values.

### Leaf expression of hormone‐related genes (RNA‐seq)

To place the transcriptomic data in a physiological context, the specific expression of genes related to major hormones (CK, ABA, JA, SA, GA and ethylene) metabolism and signalling was analysed in the leaves (Jetha et al., 2014; Larriba et al., 2021). Over 400 hormone‐related genes, only a limited number of differentially expressed genes were detected in the basal comparisons among rootstocks under control conditions (C1–C3) and in the salinity comparisons between rootstocks (C4–C6), with C1 and C4 showing only 1 and 25 significant DEGs, respectively, whereas the remaining contrasts showed no significant differential expression. Therefore, subsequent analyses focused on the most biologically informative contrasts, namely C7, C8 and C9 (control versus salinity within each graft combination), where the strongest transcriptional responses were observed. Particular attention was given to genes associated with the favourable phenotype of *ACCD* rootstocks (C9) in comparison with WT and *epi* grafts (C7 and C8) (Figure 4A; Table 4, Tables S6–S10 and S12–S14).

Among sixty‐two genes related to ethylene biosynthesis and signalling (Table S6), *ACO1* and *ACO2* biosynthesis genes (Solyc07g049530, Solyc12g005940) were upregulated by salinity regardless of the graft combination (Figures 1E and 5; Table 4, Table S6), as mentioned above. In contrast, *ACS12* and *ACS10* genes (Solyc08g079750, Solyc03g007070) were strongly induced by salinity, especially in the leaves of *epi* grafts (Figure 5; Table 4, Table S6).

**Figure 5:**
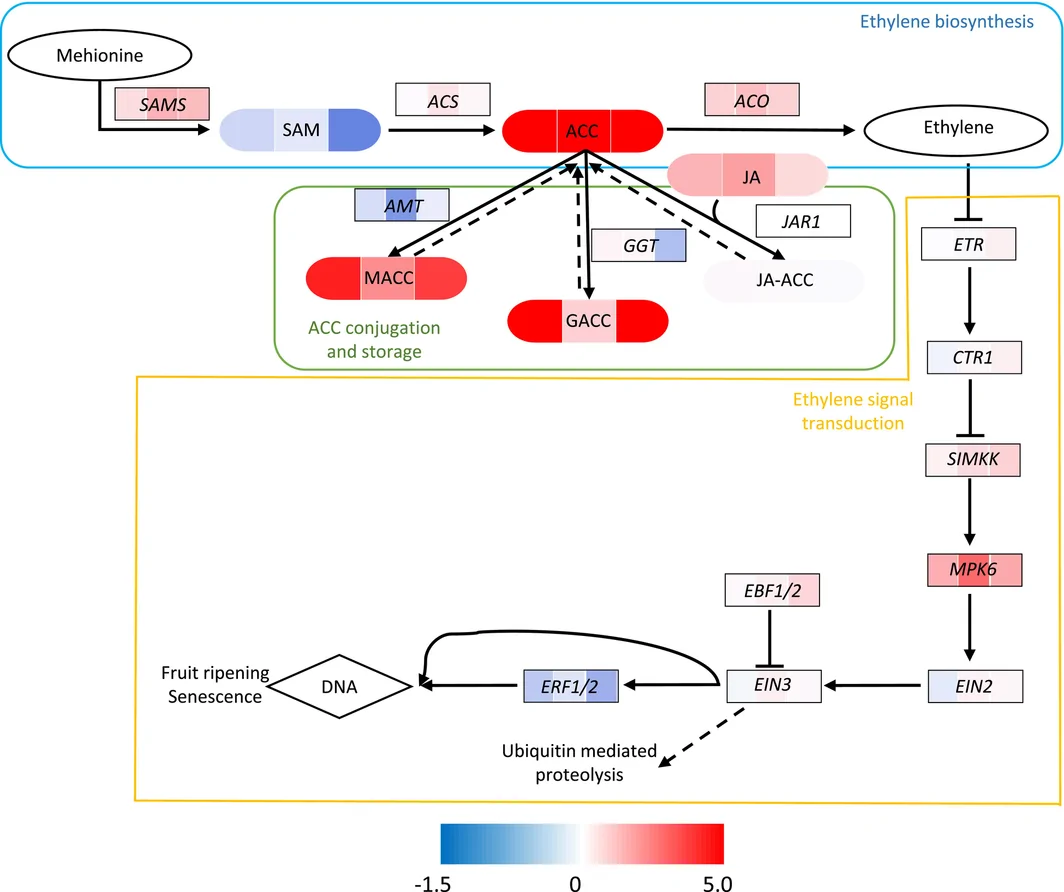
Diagram showing salt stress‐induced alterations in the ethylene metabolic pathway in leaves of tomato plants grafted onto different rootstocks, based on three contrasts control versus salt: C7 (WT), C8 (*epi*) and C9 (*ACCD*). The pathway is divided into three main sections: ethylene biosynthesis (blue box), ACC conjugation and storage (green box) and ethylene signal transduction (yellow box). Metabolites are represented as semicircles, while genes are represented as rectangles. Each element is subdivided into three sections corresponding to the different contrasts: left (C7), centre (C8) and right (C9). The colour code corresponds to the heatmap analysis of log2‐fold change (log2FC), as detailed in Table S6, with red indicating a log2FC of +5.0 and blue indicating −1.5.

Concerning ABA metabolism, salinity downregulated several *NCED* isoforms (Solyc09g016720, Solyc07g056570), particularly in *epi* and *ACCD* grafts (Figure 6; Table 4, Table S7). In contrast, two genes associated with ABA catabolism (CYP707A1/A3; Solyc04g078900, Solyc08g005610) were significantly upregulated in response to salt, especially in *epi* and *ACCD* rootstocks. For ABA signalling, PYL5 (Solyc09g015380) was salt‐induced in *ACCD* plants, and downregulated in *epi*, while *PP2C* (Solyc03g096670), a negative regulator of *PYL5*, was induced in WT and *epi* grafts. Additionally, *SnRK2*, which is negatively regulated by *PP2C*, comprises several isoforms, most of which were downregulated under salt treatment, especially in the epi grafts, except for 2 isoforms (Solyc05g056550 and Solyc02g090390) that were upregulated in *ACCD* grafts (Figure 6; Table 4, Table S7).

**Figure 6:**
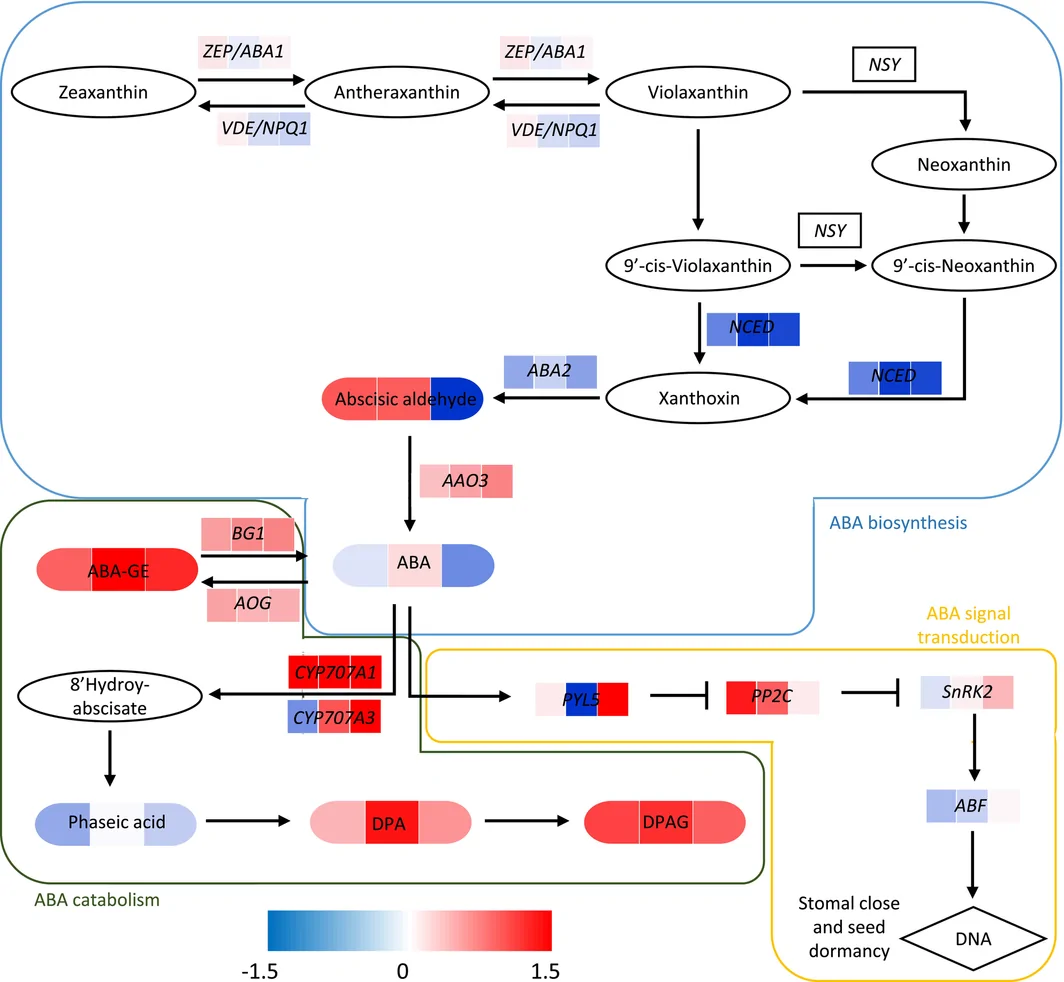
Diagram showing salt stress‐induced alterations in the ABA metabolic pathway in leaves of tomato plants grafted onto different rootstocks, based on three contrasts control versus salt: C7 (WT), C8 (*epi*) and C9 (*ACCD*). The pathway is divided into three main sections: ABA biosynthesis (blue box), ABA catabolism (green box) and ABA signal transduction (yellow box). Metabolites are represented as semicircles, while genes are represented as rectangles. Each element is subdivided into three sections corresponding to the different contrasts: left (C7), centre (C8) and right (C9). The colour code corresponds to the heatmap analysis of log2‐fold change (log2FC), as detailed in Table S7, with red indicating a log2FC of +1.5 and blue indicating −1.5.

As previously stated, ABA metabolism seems to play a key role in the resilience of plants grafted onto *ACCD* rootstocks under salt stress. To further explore gene‐metabolism relationships within this pathway, we conducted a network analysis integrating expression and metabolite levels from leaves of *ACCD* grafted plants (Figure S6), which identified the transcription factors *E2L, WRKY40b, ABF1, ABF2* and *ABF3* as key regulators of the responses of *ACCD* grafts to salt stress.

For GAs, a significant upregulation was observed in one isoform of *GA2ox3* (Solyc07g061730) and two isoforms of *GID1* genes (Solyc09g074270, Solyc06g008870), involved in GA catabolism and signal transduction, respectively, across all graft combinations (Table S8). Furthermore, three isoforms of *DXS1* (Solyc11g010850, Solyc01g067890, Solyc08g066950), one isoform of *GA20ox2* (Solyc01g093980), and *GA3ox2* (Solyc06g066820) displayed a strong upregulation mainly in the *ACCD* rootstocks. More than 15% of CK‐related genes exhibited a significant deregulation under salinity (Table 4, Table S9), and their expression varied among rootstock genotypes in response to salinity. *LOG3* (Solyc04g081290) and *LOG5* (Solyc08g062820), both involved in CK biosynthesis, were upregulated in *epi* grafts under salinity, whereas *IPT5* (Solyc11g066960), another CK biosynthesis gene, together with several glycosyltransferase genes (*ZOGs*) involved in CK homeostasis, were more highly expressed in *ACCD* grafts under salinity (Table 4, Table S9). Likewise, several CK‐signalling genes, including *AHPT1L* and type‐B response regulators (*ARR‐B*), were induced in plants grafted onto *ACCD* rootstocks, whereas several type‐A response regulators (*ARR‐A*) were downregulated (Table 4, Table S9). Finally, in *ACCD* grafts exposed to salinity, leaf expression of JA biosynthetic genes, including *LOX6, LOX2* and *AOC1/2/3/4* (*Solyc08g014000, Solyc03g122340, Solyc02g085730*), as well as the signalling regulator *JAZ1* (*Solyc07g042170*), was markedly enhanced (Table 4, Table S10). By contrast, the JA biosynthetic gene *JAR1* (*Solyc07g054580*) and the JA receptor *JAZ2* (*Solyc12g009220*) had elevated expression in WT and *ACCD*, but not in *epi* grafts (Table 4, Table S10).

## DISCUSSION

Rootstock‐specific production of the ethylene‐precursor ACC significantly affected its concentration in the scion (Figure 1A,B), particularly under salinity, when root‐derived ACC is transported to the shoot, as reflected by the ACC levels detected in leaf xylem sap (Figure 1C, Albacete et al., 2008), influencing physiological and agronomic responses to stress (Table 1), through local (RSA) and systemic (nutritional, metabolomics and transcriptomic) mechanisms. Consistent with ACC levels, expression of ethylene biosynthetic genes *ACO1* and *ACO2* was constitutively higher and stress inducible in *epi* roots, but lowest and unaffected by the stress in *ACCD* rootstocks, while leaf expression remained similar for *ACO1* among grafts, and salinity induced *ACO2* to a higher extent in *epi* ones (Figure 1G,H, Figure S5). These results suggest a differential ACC‐mediated and tissue‐dependent induction of ACO isoforms and subsequent ethylene production (Houben et al., 2026). The levels of ACC conjugates and the precursor SAM are also consistent with ACC concentrations in different grafts (Figure S2a). Particularly, the elevated GACC levels in *epi* leaves indicate an increased ACC transport in the xylem (Vanderstraeten & Van Der Straeten, 2017), as reflected by the ACC concentration in the leaf xylem sap (Figure 1C). Since ACC synthesis from SAM is the rate‐limiting step in ethylene biosynthesis (Park et al., 2018; Yang, 1980) and the enzyme converting ACC to ethylene is largely constitutive (Fujino et al., 1988; Yang & Hoffman, 1984) and substrate/stress inducible (Houben et al., 2026; Figures 1E,F and 5; Figure S5), these results are consistent with a role for the rootstock‐to‐scion ACC transport in ethylene‐mediated stress responses (Albacete et al., 2008; Dodd, 2005; Pérez‐Alfocea et al., 2010), which was higher in *epi* and reduced in *ACCD* grafts (Figure 5). However, a direct role of ACC as a stress sensor and root‐to‐shoot signal cannot be excluded (Ghanem et al., 2008; Martínez‐Andújar et al., 2016, 2017; Mou et al., 2020, 2025; Vanderstraeten et al., 2019).

The rootstock‐sourced ACC/ethylene was associated with differences in local (RSA) and systemic (through root‐to‐shoot communication) developmental, nutritional and hormonal responses (Albacete, Martínez‐Andújar, & Pérez‐Alfocea, 2014; Dodd, 2005; Martínez‐Andújar et al., 2021; Pérez‐Alfocea et al., 2010). While only slight differences in root architecture were observed in the absence of stress, salinity increased the number, length and volume of LR in WT and *ACCD* plants, but not in *epi* grafts (Figure 1I), indicating that these RSA responses to salt stress were conserved in grafted plants with respect to young whole seedlings (Figure S1). These results are consistent with ACC levels in the roots and the leaf and flower ionomes, with *ACCD* and *epi* grafts showing the highest and lowest nutrient concentrations under salinity, respectively (Table 2).

*ACCD* grafts enhanced nutrient uptake and accumulation in the scion, particularly P, Ca and S (Table 2), improving leaf physiology and stress tolerance. P nutrition is a key factor in salt tolerance (Su et al., 2022) since its deficiency alters source–sink relations, mainly reducing sink number and activity (Chapin III & Wardlaw, 1988). Under salinity, leaf P decreased by 20% in *epi* and 50% in WT grafts but increased by 20% in *ACCD* plants, consistent with induced cellular responses to P starvation (Figure 4G,H; Figure S4a) and with photosynthetic, growth and yield‐related parameters under salinity (Table 1). Despite similar RSA between *ACCD* and WT grafts, the higher shoot P concentrations in *ACCD* likely reflect enhanced P transport or metabolism rather than RSA traits. Three tomato phosphatases (*SPX3*, *PAP17* and *PEPC1*) were significantly upregulated in salinised *ACCD* leaves (Figure 4H), supporting adaptive regulation of P uptake, transport, storage and homeostasis, driving essential adaptations to P availability (Bhadouria & Giri, 2022; Hanchi et al., 2018; Wang et al., 2024). This agrees with earlier findings where low‐ACC rootstocks promoted shoot and root growth, and nutrients and bioactive cytokinin (*t*Z) transport to the scion under low K (Martínez‐Andújar et al., 2016) and P (Martínez‐Andújar et al., 2017) nutrition, compared to high ACC‐sourced rootstocks. Moreover, *ACCD* rootstocks decreased Na transport to the scion (Table 2), mitigating salt toxicity in the leaves (Martínez‐Andújar et al., 2016, 2021; Pérez‐Alfocea et al., 2010), which is also consistent with the GA_3_ increase in leaves of *ACCD* grafts (Achard et al., 2006; Shahzad et al., 2021), and the differential gene expression related to Na transport and homeostasis (Figure S4b).

While Ca translocation to sink organs is typically restricted (de Bang et al., 2021), stress‐induced Ca waves from roots or leaves toward flowers can act as systemic signals under heat or osmotic stresses, mediated by JA and ethylene (Bisht et al., 2025; Grenzi et al., 2023). Salt‐induced JA accumulation occurred only in roots and leaves of WT and *epi* grafts (Figure 2), while biosynthetic (*LOX6, LOX2*, *AOC1/2/3/4*) and signalling (*JAZ1*) genes were strongly induced in leaves of *ACCD* grafts (Table 4, Table S10). Conversely, the receptor *JAZ2* and the biosynthetic *JAR1*, essential for JA signalling and stress protection (Staswick & Tiryaki, 2004), were unaltered or suppressed in *epi* leaves, respectively, while both were induced in the other grafts. Interestingly, the lack of JA response to salinity in *ACCD* roots and leaves agrees with stabile Ca levels in leaves and flowers compared to WT and *epi* grafts (Figure 2; Table 2). Therefore, Ca translocation from leaves to flowers may signal stress sensitivity, influenced by the rootstock genotype and its ACC/ethylene production capacity. Overall, reduced rootstock‐sourced ACC under salinity was associated with improved P, Ca, S and Na homeostasis in leaves and flowers through hormonal and transcriptomic reprogramming, which may contribute to yield stability under salinity through both local (RSA) and systemic (nutrient and Na homeostasis) mechanisms. Similarly, while only minor phenotypic differences were observed in the shoot under control conditions, systemic responses to salinity also depended on the rootstock genotype (Table 1). *ACCD* grafts exhibited a more vigorous vegetative growth and higher and stable yield under salinity, whereas *epi* rootstocks performed better than WT under control but were the most affected by the stress in fruit weight and total biomass production (Table 1). Enhanced performance of *ACCD* grafts under salinity was associated with improved photosynthetic parameters, higher FN and FW and increased truss branching (Table 1), linked to *SEP4* gene upregulation (Figure 4H), involved in inflorescence meristem identity by interacting with hormones like GAs, CKs and auxins (Burgess, 2017; Ditta et al., 2004; Jetha et al., 2014). For instance, the increased GA_3_ concentration in leaves and flowers of salinised *ACCD* grafts (Figure 2) matched the upregulation of GA biosynthesis genes (*GA20ox* and *GA3ox*) (Table S8) and the promotion of vegetative and reproductive growth (e.g. *SEP4*), and supports the ethylene‐GA interaction for adapting leaf development to environmental changes (Skirycz et al., 2011). Under salinity, *ACCD* grafted plants also exhibited thicker leaves, epidermis and PP (Figure 3B–I), supporting photosynthetic efficiency, stable stomatal conductance (Table 1) and improved leaf water status (Acosta‐Motos et al., 2017; Geissler et al., 2009; Liu et al., 2022). ACC can directly alter leaf anatomy traits through modifying cell wall composition and properties (Tsang et al., 2011; Xu et al., 2008). Overall, besides RSA, low root ACC production in *ACCD* grafts also improved photosynthesis, plant vigour and yield under salinity, compared to normal (WT) and high (*epi*) ACC‐sourced rootstocks, highlighting that reduced ACC favoured growth maintenance under salt stress (Albacete et al., 2008; Albacete, Cantero‐Navarro, et al., 2014; Albacete, Martínez‐Andújar, & Pérez‐Alfocea, 2014; Bhise & Dandge, 2019).

In addition, upregulation of several steroid biosynthesis‐related genes in *ACCD* grafts under salinity (Figures S3e,f and S4c) likely improved chlorophyll status (SPAD and *Fv/Fm*) and photosynthesis (*A*_*N*_, *g*_*S*_, *E*) by reinforcing membranes (Kumar et al., 2015), preventing water loss (Rogowska & Szakiel, 2020) and protecting the photosynthetic apparatus (Guo et al., 2019). Reduced leaf ABA levels in salinised *ACCD* grafts (Figure 2; Table 3) matched higher *g*_*S*_ and *A*_*N*_ and were consistent with both metabolomic and transcriptomic data indicating reduced ABA biosynthesis and enhanced catabolism. The decrease in leaf ABA levels in salinised *ACCD* grafts is supported by reduced ABA aldehyde and increased ABA catabolites and conjugated forms (DPA, DPAG, ABA‐GE) (Table 3). These metabolomic changes agree with the strong downregulation of ABA biosynthesis *NCED3* gene and the induction of the ABA 8′‐hydroxylase genes *CYP707A1/3* (Kushiro et al., 2004) (Figure 6, Figure S6; Table S7). In tomato, low ACC and high ABA/ACC ratio in the leaves have been associated with plant growth and fruit yield under salinity (Albacete et al., 2008, 2009; Ghanem et al., 2008; Cohen et al., 2010; this study). Besides, several ABA signalling (e.g. *WRKY40* and *PYL5*) and *ABC* transporters were upregulated in salt‐treated *ACCD* leaves, whereas the negative regulator *PP2C* (Bai et al., 2021; Li, Liu, Ruan, et al., 2020) was upregulated in the leaves of WT and *epi* grafts (Table 4, Table S7). Upregulation of *WRKY40*, linked to salt tolerance in *Arabidopsis* (Li, Liu, Ruan, et al., 2020), together with the lack of ABA accumulation in the roots and the higher DPAG levels (Table 3), suggest that reduced root‐to‐shoot ABA transport may also account for the lower leaf ABA in *ACCD* grafts under salinity. Overall, while leaf ABA metabolism in *ACCD* favoured lower hormone accumulation, ABA signalling remained active, contributing to stress adaptation (Martínez‐Andújar et al., 2020; Rehman et al., 2021), while preserving stomatal conductance and photosynthesis.

CKs also mediate stress responses through interaction with ethylene (Liu et al., 2020; Nguyen et al., 2021), thereby contributing to the regulation of source–sink relations. Leaf CK levels (Figure 2) and CK‐related gene expression (e.g. *LOG5* and *IPT5*) (Table 4, Table S9) varied according to the rootstock genotype under salinity. In *epi* grafts, the changes in the bioactive CKs *t*Z, ZR and iP were associated with *LOG3* induction (Table 4, Table S9), whereas CKs levels remained stable in *ACCD* grafts, together with upregulation of *ZOG* and *AHPT1L*, genes involved in CK homeostasis and signalling through glycosylation (Hallmark et al., 2020; Šmehilová et al., 2016) and phosphorylation of ARR‐B response regulators, respectively (Argyros et al., 2008; Ishida et al., 2008), and previously associated with drought and salt tolerance in rice (Li, Liu, Li, et al., 2020; Sun et al., 2014). Likewise, the contrasting regulation of *ARR‐B Response Regulators* and *Cytokinin Response Factors* (*CRFs*) supports an ACC/ethylene modulation of CK signalling, as reported under cold stress (Jeon et al., 2010; Shi et al., 2012), likely enhancing salinity tolerance. Cytokinin Response (*CRF2, CRF4*) were induced in *ACCD* grafts under salinity, while *CRF4* was downregulated and *CRF6* upregulated in *epi* leaves (Table 4, Table S9). *CRFs* are involved in osmotic regulation, antioxidant and PSII efficiency under salinity (Keshishian, 2018; Qin et al., 2017). Hence, the salt‐induction of *MYB‐like/ARR2‐like* transcription factor *ARR‐B11* in *epi* grafts is consistent with flavonoid (e.g. taxifolin) accumulation (Nakabayashi et al., 2014; Pan et al., 2013; Ye et al., 2015) (Table 4, Tables S3 and S11). However, the specific induction of the K transporter *SlHAK7* in *ACCD* leaves (Table S5), together with the reported regulation of *AtHKT1;1* by *ARR1‐12* (Mason et al., 2010), is consistent with the improved K/Na homeostasis and reduced Na accumulation observed in *ACCD* plants (Wang et al., 2020). Thus, although both contrasting ACC conditions activated adaptive CK‐related responses, low root‐derived ACC preferentially promoted CK homeostasis and signalling associated with growth maintenance (*ZOG, AHPT1L, ARR‐B5/12, CRF2/4*), whereas high ACC favoured alternative signalling pathways linked to stress protection and flavonoid accumulation (*ARR‐B11, CRF6*). The putative relationship between root ACC/ethylene levels and iP, *LOG3*, *ARR‐B11* and flavonoid accumulation in leaves deserves further investigation. Overall, rootstock‐mediated ACC production interacts with CK homeostasis and signalling, influencing growth and physiological adaptive responses to salinity in the scion variety.

As a consequence of hormonal and transcriptomic reprogramming, leaf carbon metabolism was also altered in both *epi* and *ACCD* grafts (Figure 4E,F; Figure S3d,e). *ACCD*‐grafted plants maintained active source–sink relationships under salinity, with stable sucrose and hexoses levels in leaves and flowers, while these sugars decreased in WT and *epi* grafts (Figure 3A). Leaf starch decreased in WT and *ACCD*, while increased in *epi* grafts (Figure 3A), consistent with starch synthase gene induction in *epi* leaves (Table S5). Since starch remobilisation supplies carbon and energy when photosynthesis is limited (Thalmann & Santelia, 2017), this response appears to be operative in WT, impaired in *epi and* unnecessary in *ACCD* grafts, aligning with their contrasting leaf anatomy (Figure 3B–I). *ACCD* grafts also maintained higher or more stable levels of several amino acids concentrations in flowers under salinity (Table S4), including amino acids involved in flower development (Trp and Tyr) and yield (Borghi & Fernie, 2017), maintaining cell turgor (Pro and Hyp) and withstanding stressful conditions (Kahlaoui et al., 2018), as well as attracting pollinators (Murray et al., 2025), favouring flower pollination and fruit set, as observed in *ACCD* grafts (Table 1). Taken together, these findings suggest that *ACCD* rootstocks promoted a better maintenance of photoassimilate and source–sink management under salinity, supporting plant growth and yield, while *epi* rootstocks favoured the accumulation of flavonoids, potentially as an adaptive mechanism to mitigate the effects of salt stress. These results support a balancing role of ethylene in abiotic stress responses between plant growth and survival (Albacete, Cantero‐Navarro, et al., 2014; Chen et al., 2021; Pierik et al., 2006) through the regulation of source–sink relations (Figure 7).

**Figure 7:**
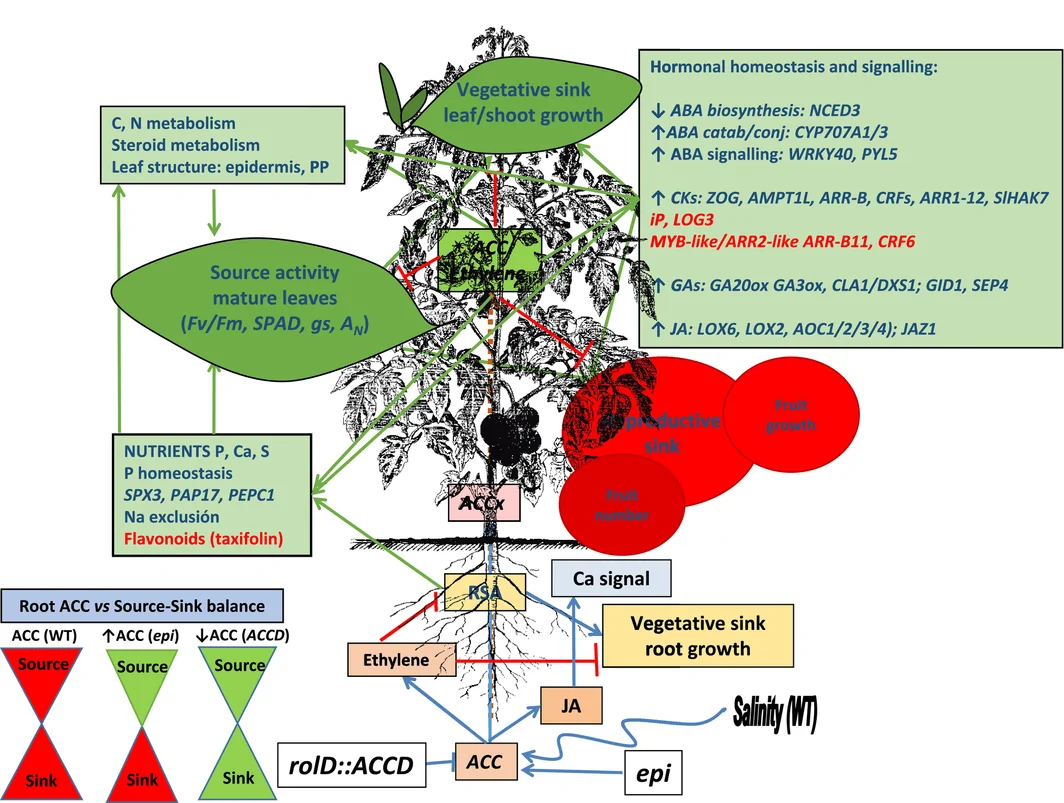
Proposed physiological model explaining the impact of the root‐sourced ACC in local and systemic responses influencing plant growth and yield under saline stress. Normally (WT), salt stress in the rootzone induces ACC (and ethylene) synthesis locally altering root development, while ACC may also be transported to the shoot through the xylem (ACCx), contributing to systemic alteration in source–sink relations and leading to reduced photosynthesis, growth and yield (reduced source and sink activities). Low root ACC production in *ACCD* grafts firstly favours RSA adaptive responses to stress and reduces the ACC evolution in the aerial organs. Compared to normal (WT) and high (*epi*), low root ACC improved leaf nutrient status through RSA (nutrient uptake and Na exclusion) and homeostatic processes (e.g. P) leading to nutrient stability and low Na‐derived toxicity. Moreover, low‐ACC also interacted with leaf hormones, reducing ABA levels but enhancing ABA signalling that favours adaptive responses while preserving gas‐exchange and photosynthesis. Low ACC also promoted CK, GA and JA homeostasis and signalling favouring growth and adaptive processes in vegetative and reproductive tissues (maintain source and sink activities). However, high ACC induced ethylene biosynthetic genes and inhibited RSA responses negatively influencing leaf nutrient status, but also induced some adaptive responses such as the accumulation of protective flavonoids in leaves leading to preserving leaf growth and photosynthesis, while strongly inhibited the fruit growth (maintain source activity but reduced reproductive sink activity). Arrow and bar heads indicate positive and negative regulation, respectively. Green arrows and blue text indicate activated processes in *ACCD* plants, while red text indicates activated processes in *epi* grafts. Green and red triangles indicate positive and negative regulation of source and sink activities in different ACC‐sourced rootstocks, respectively.

In conclusion, root‐sourced ACC modulates vegetative and reproductive growth in the scion under salt stress through acting on source–sink relations. While stress‐induced ACC reduced both source (*A*_*N*_, *Fv/Fm*, SPAD) and sink activities (leaf and fruit growth) in WT grafted plants, high ACC in *epi* inhibited sink activity in those organs, mainly in the fruits, while protected source activity in mature leaves in a survival‐like strategy (Figure 7). However, a low ACC production in *ACCD* grafts maintained both source and sink activities in mature source leaves and vegetative and reproductive sink organs, resulting in sustained plant growth and yield under salinity, consistent with a role of the root‐sourced ethylene‐precursor ACC in modulating this stress (Albacete et al., 2008; Albacete, Cantero‐Navarro, et al., 2014; Albacete, Martínez‐Andújar, & Pérez‐Alfocea, 2014; Bhise & Dandge, 2019). The improved performance of *ACCD* rootstocks resulted from coordinated local and systemic mechanisms under salinity, resulting in enhanced root system, diminished Na toxicity in shoots and improved leaf P, Ca and S nutrition, photosynthesis and source–sink relations, together with hormonal reprogramming characterised by reduced leaf ABA and increased activity of growth‐promoting and anti‐senescing hormones CKs and GAs. These physiological responses were accompanied by transcriptomic signatures consistent with enhanced P metabolism, Na homeostasis, sterol biosynthesis, ABA catabolism and CK‐ and GA‐related signalling pathways (Table S15). Conversely, high ACC production in *epi* rootstocks also activated stress‐adaptive responses, including flavonoid accumulation and alternative hormonal signalling mechanisms. Although a central role for ethylene as the final downstream and major actor has not been proven nor ruled out in this study, the results support that the promotion of resilient physiology and/or stable yields in a tomato variety grown under variable salinity through modulating root production of its precursor ACC is of particular relevance for rootstock and crop breeding. Moreover, the Micro‐Tom genetic toolkit used in this study eases the practical/commercial implications of this study given the high genetic compatibility and performance of grafts with elite varieties observed in long‐term field experiments.

## MATERIALS AND METHODS

### Plant material

To study the effects of root‐derived ethylene on tomato salinity response, the ethylene‐overproducing mutant *epinastic* (*epi*) and a transgenic line overexpressing the gene encoding for the enzyme ACCD under the root‐specific *rolD* promoter (*roID::ACCD*) of *Agrobacterium rhizogenes*, both on the Micro‐Tom (MT) background, were used as rootstocks of the commercial tomato cherry variety UniDarkwin (UD; Unigenia Semillas, Murcia, Spain). The root‐specific overexpression of *ACCD* in transgenic plants reduces ethylene production by over 90% compared to wild‐type plants (Klee et al., 1991). The mutant *epi* is a BC6Fn introgressed LA2089 (cv. VFN8) MT line (Carvalho et al., 2011) harbouring a pleiotropic mutation leading to ethylene overproduction in root and shoot tissues that activate ethylene responses (Fujino et al., 1988), although not in a whole plant constitutive way (Barry et al., 2001). Higher ethylene concentrations in *epi* resulted from greater 1‐aminocyclopropane‐1‐carboxylic acid content rather than increased ethylene forming enzyme activity (Fujino et al., 1988). Since the *epi* roots overproduce ACC prior to ethylene conversion and the ethylene‐phenotype is mainly manifested in the shoot (Barry et al., 2001; Fujino et al., 1988), the *epi* mutant is an adequate counterpart of *ACCD* to study the impact of root‐sourced ACC in grafting experiments. Indeed, the *epi*‐grafted plants did not show the typical epinastic growth traits (this study) observed in the whole plants (Carvalho et al., 2011).

Seeds were germinated in vermiculite under controlled conditions (26–28°C, 80–90% relative humidity) in the dark and grafted at the 2–3 true‐leaf stage using the splicing method (Savvas et al., 2011). Grafting with *epi* mutant and *ACCD* transgenic line, along with the wild‐type MT (WT) used as a control, resulted in three graft combinations: UD/*epi*, UD/*ACCD* and UD/WT. Forty days after grafting, plants were transferred to a commercial‐type plastic greenhouse (south‐eastern Spain) and cultivated in sand substrate during the spring–summer season (Martínez‐Andújar et al., 2023). The experiment followed a factorial design with three graft combinations (UD/WT, UD/epi and UD/ACCD), two treatments (control and salinity) and six biological replicates per graft combination and treatment (3 × 2 × 6 = 36 plants in total). Plants were randomly distributed, and drip‐fertigated.

From 30 days after transplanting, 50% of the plants from each graft combination (n = 6) were subjected to salinity by adding 75 mM NaCl to the nutrient solution, resulting in an electrical conductivity (EC) of 8.9 dS m^−1^ and was maintained for a period of 14 weeks (saline treatment, ST). The remaining half of the plants received the nutrient solution (EC = 3 dS m^−1^) for the whole period (control treatment).

### Plant phenotyping

Measurements were performed using three to six plants per graft combination and treatment, depending on the analysis. The sampling timeline was as follows: gas exchange and chlorophyll fluorescence at 10 weeks of salt treatment (WST) (*n* = 3–6, three technical readings per plant); leaf anatomy at 13 WST (*n* = 3); shoot and root biomasses, stem diameter, leaf fresh weight and area, flower and leaf ionome analyses, flower, leaf and root hormones and metabolites, and leaf starch concentration at 14 WST (*n* = 6); RNA‐seq analysis at 14 WST (*n* = 3) and qPCR analysis in leaves at 14 WST (*n* = 4).

Total yield was determined by collecting all fruits from each individual plant throughout the harvest period. Fully ripe fruits were harvested weekly for 2 months, and fruit number (FN), fruit weight (FW) and truss length (TL) were recorded. At the end of harvest (19 weeks after transplanting, 14 WST), shoot (SFW) and root (RFW) biomasses were determined, and stem diameter (SD) at the second node was measured using an electronic digital Vernier calliper (0–150 mm). Young fully expanded leaves, roots and flowers were sampled, frozen in liquid nitrogen and stored at −80°C for hormonal and gene expression analysis. Leaf xylem sap was obtained according Albacete et al. (2009).

After 10 WST, photosynthesis (*A*_*N*_), stomatal conductance (*g*_*s*_) and evapotranspiration (E) were measured on fully expanded young leaves using a CIRAS‐2 (PP Systems, MA, USA) between 09.00 and 12.00 h, at 400 ppm CO_2_ and 1500 μmol m^−2^ s^−1^ photosynthetic active radiation (PAR). Chlorophyll fluorescence was recorded in dark‐adapted (15 min) leaves using a chlorophyll fluorometer OS‐30 (OptiSciences, Herts, UK) with an excitation intensity of 3000 μmol m^−2^ s^−1^. Chlorophyll content (SPAD) was determined using a portable chlorophyll meter SPAD‐502 (Konica Minolta, Tokyo, Japan). For each genotype and treatment, three readings were taken per leaf on different plants, and the mean value for each plant was used for analysis. At 14 WST, the second fully expanded mature leaf above the fourth truss was collected to determine leaf fresh weight (LFW) and leaf area (LA) using an LI‐3100 AC area meter (LI‐Cor, Lincoln, USA).

### Root system architecture (RSA) and ethylene analysis in non‐grafted seedlings

An additional experiment was conducted using non‐grafted tomato seedlings of MT‐WT, *epi* and *ACCD* genotypes grown under controlled growth chamber conditions to evaluate root system architecture (RSA) responses under salinity. Plants were cultivated for 30 days in a growth chamber under a 16 h light/8 h dark photoperiod at 28°C/18°C (day/night). Control plants were irrigated with Hoagland nutrient solution, while salinity‐treated plants received the same solution supplemented with 75 mM NaCl. At the end of the experiment, 4–13 roots were carefully collected and scanned using an Epson Expression 11000XL scanner (Epson, Japan). Root system architecture traits were quantified using WinRHIZO Pro 2016 software (Regent Instruments Inc., Quebec, Canada).

Ethylene production was determined as described previously by Álvarez‐Hernández et al. (2020). Briefly, intact seedlings were placed in hermetically sealed glass jars for 2 h to allow ethylene accumulation. Gas samples (1 mL) were collected from the headspace using a gas‐tight syringe and injected into a gas chromatograph (Clarus 500 GC; PerkinElmer Inc., Shelton, CT, USA) (Álvarez‐Hernández et al., 2020). Two technical replicates were performed per sample. Ethylene concentrations were quantified using external standards and expressed as nmol g^−1^ h^−1^.

### Pollinator preference quantification

Although UD is a self‐pollinated variety, managed bumblebees (*Bombus terrestris*) were used to ensure pollination in the whole greenhouse area, following established commercial protocols. Each hive (~ 50 workers) can pollinate between 300 and 500 plants over 8–12 WST. Pollinators' preferences were monitored weekly across the plant's entire reproductive phase, using pre‐marked flower trusses for tracking. The percentage of flowers visited by bumblebees (%FVB, Table 1) was recorded following Martínez‐Andújar et al. (2023).

### Ionome analyses

The same leaves collected for LA and LFW analysis at 14 WST, and flowers sampled during the same period, were dried (48 h, 80°C), milled to a powder, and 100 mg (flower and leaf) of dry tissue were digested using a HNO_3_:HClO_4_ (2:1, vol/vol) solution. Samples were analysed by inductively coupled plasma spectrometry (ICP‐OES, Thermo ICAP 6000 Series). Total C and N contents were determined by the combustion method using an elemental analyser (LECO TRUSPEC, Geleen, The Netherlands).

### Hormones, soluble carbohydrates, amino acids and flavonoids quantification

The major classes of plant hormones, including CKs [trans‐zeatin (tZ), zeatin riboside (ZR) and isopentenyladenine (iP)], gibberellin A1 (GA1), A3(GA3) and A4 (GA4), jasmonic acid (JA), salicylic acid (SA) and the ethylene‐precursor ACC, ABA and its derivatives ABA aldehyde and catabolites (ABA glucosyl ester (ABA‐GE), neophaseic acid (neoPA), phaseic acid (PA), dihydrophaseic acid (DPA) and DPA glucoside (DPAG) and flavonoids (cyanidin, genistein, luteolin, quercetin and taxifolin)) were extracted and analysed using ultra‐high‐performance liquid chromatography‐mass spectrometry (UHPLC–MS) following the protocol described previously in Albacete et al. (2008) and Martínez‐Andújar et al. (2021). Additionally, amino acids and soluble sugars (sucrose, fructose and glucose) were also analysed using this method as described in Martínez‐Melgarejo (2022) and Ormazabal et al. (2024). Ten microliters of each filtered extract (leaf, root or flower) were injected into a UHPLC–MS system consisting of an Accela Series UHPLC coupled to an Orbitrap Exactive mass spectrometer (ThermoFisher Scientific, MA) using a heated electrospray ionisation (HESI) interface. Mass spectra were obtained using the Xcalibur software version 2.2 (ThermoFisher Scientific, MA). To quantify plant hormones, sugars and amino acids, calibration curves were constructed for each analysed component using standard solutions: hormones (0, 1, 10, 50 and 100 μg L^−1^), amino acids (10, 100, 500 and 1000 μg L^−^1) and sugars (10, 50, 100 and 400 μg L^−1^) from Sigma‐Aldrich (Merck Group, Germany). Flavonoids, ABA precursors and catabolites, SAM and ACC precursor and derivatives were identified by extracting the exact mass of the target catabolite from the full scan chromatogram obtained in the negative mode, adjusting a mass tolerance of ≤1 ppm. Concentrations were semi‐quantitatively determined from the extracted peaks using the calibration curve of ABA (precursors and catabolites), ACC (precursor and derivatives) or the total area (flavonoids).

### Starch analysis

Starch concentration was analysed in the same leaves used for ionomic analysis and collected at 14 WST, following the protocol described by Nicolás et al. (2015), with some modifications.

### Microscopic leaf morphometric analysis

To assess leaf structural differences among graft combinations under salinity, six square sections (1 mm^2^) were taken from the youngest fully expanded leaves of 3 plants per graft after 13 WST, avoiding the midrib. Measurements obtained from the six sections of each plant were averaged prior to statistical analysis; therefore, the biological replicate (plant) was considered the experimental unit (*n* = 3). Samples were fixed and post‐fixed following Fernández‐García et al. (2014). After embedding in Spurr resin to form blocks, they were sectioned into slices 0.5–0.7 μm thick using a Leica EM UC6 ultra‐microtome. Sections were stained with 0.5% toluidine blue, mounted in DPX and observed with a Leica CTR 6000 fluorescence microscope. The percentages of area occupied by palisade parenchyma (PP), spongy parenchyma (SP), and intercellular spaces (IS) in leaves from grafted plants were measured by the LAS AF Lite software.

### RNA isolation and RNA‐seq analysis

To study the impact of different ethylene‐producing rootstocks on leaf gene expression under control and saline conditions of 14 WST, total RNA was extracted from approximately 100 mg of powdered leaf and root tissue using NBS Spin Column All‐in‐one Miniprep (NBS Biologicals, UK) following the manufacturer's protocol. The extracted RNA was subsequently stored at −80 °C. RNA integrity was confirmed using a 2100 Bioanalyzer (Agilent Technologies, USA). RNA from leaves was sequenced (paired‐end, 100 bp) using the BGISEQ‐500 pipeline (BGI‐Tech, China). Clean reads were obtained using SOAPnuke and evaluated using FastQvalidator. For bioinformatics analysis, the workflow described in Larriba et al. (2021) was followed. Briefly, clean RNA‐Seq reads were mapped to the *S. lycopersicum* genome build SL4.0 (Larriba et al., 2021) using STAR 2.7 (Hosmani et al., 2019). Gene count was performed using feature Counts from the Subread package (Liao et al., 2019) with the ITAG4 gene models from SolGenomics. For the statistical treatment, the web‐tool ShinyGO 0.8 (Ge et al., 2020) was used to perform GO enrichment analysis. Putative tomato orthologues from *A. thaliana* were identified using Proteinortho software (Lechner et al., 2011). The analyses for heatmap and hierarchical clustering were performed using Morpheus.

### First‐Strand cDNA synthesis and qRT‐PCR

Retrotranscription was performed using 5 μg of RNA with the RevertAid First Strand cDNA Synthesis Kit (Thermofisher). Quantitative PCR (qPCR) was performed with 20 ng of cDNA and PyroTaq EvaGreen qPCR Master Mix on a StepOne Real‐Time PCR System (Thermofishser), including ROX as a passive reference. Transcript levels of genes *TAS14*, *PIP1.2*, *CYP707A1*, *ACO1 and ACO2* were quantified, with primers listed in Table S1. Relative expression was calculated following Pfaffl (2001) and normalised to the housekeeping genes *actin*, *EF* and *GAPDH*.

### Statistical analysis

Data from phenotypic, ionomic and metabolomic analyses were subjected to two‐way ANOVA to assess the effects of genotype, treatment and their interaction, and means were compared using Tukey's test (*P* < 0.05). Correlation among variables was performed using SPSS v22.0 (SPSS Inc., Chicago, USA). For RNA‐seq, read count normalisation and differential expression were performed using DESeq2, integrated into the Differential Expression and Pathway analysis (iDEP 96) web application (Ge et al., 2018). Differentially expressed genes (DEGs) were identified using a False Discovery Rate (FDR) < 0.05 and fold change > |2|. Venn diagrams (C7‐C9; Figure 4C,D) illustrated shared and unique gene responses to salinity among genotypes.

## AUTHOR CONTRIBUTIONS

FP‐A: Conceived and designed the research. JAM‐R and CM‐A: Performed all stress experiments. EL and JG: Conducted bioinformatic analyses of transcriptomic data. JMP‐P: Executed the qPCR validation. EO and JAM‐R: Performed the microscopy analyses. LEPP: developed the mutant and transgenic lines and provided the seeds. JAM‐R, PAM‐M, ASP, JJG and CM‐A: Performed the physiological analysis. JAM‐R and PAM‐M: Carried out the hormone profiling analysis. JAM‐R: Performed the data analysis. JAM‐R, CM‐A and FP‐A: Wrote the original draft. JAM‐R, JG, CM‐A and FP‐A: Reviewed and edited the final manuscript.

## CONFLICT OF INTEREST

The authors declare no conflicts of interest.

## Supporting information


**Figure S1.** Effects of altered ACC/ethylene status on root system architecture (RSA) in non‐grafted tomato seedlings under salinity. (a) Representative root scanner images of WT, *epi* (high ethylene production) and *ACCD* (low ethylene production) tomato plants grown under control and salt (75 mM NaCl) conditions for 30 days in a growth chamber. (b) Ethylene production in WT, *epi* and *ACCD* plants grown under control and salinity conditions. Bars indicate mean values ± SE. Different letters indicate significant differences among genotypes (*n* = 3; *P* < 0.05) assessing within and between treatments. Results of two‐way ANOVA (*P* values reported) for genotype (G), treatment (T) and their interaction (G × T) are indicated in the top right of the panel. *** indicate statistically significant difference at *P* < 0.001. (c–g) Quantitative RSA parameters determined using WinRHIZO Pro 2016 software, including traits associated with primary and secondary root development. Bars indicate mean values ± SE. Different letters indicate significant differences among genotypes (*n* = 4–13, *P* < 0.05) assessing within and between treatments. Results of two‐way ANOVA (*P* values reported) for genotype (G), treatment (T) and their interaction (G × T) are indicated in the top right of the panel. ** and *** indicate statistically significant difference at *P* < 0.01 and *P* < 0.001, respectively.

**Figure S2.** ACC precursors and derivatives. (a) S‐adenosyl‐L‐methionine (SAM), (b) glutamyl‐ACC (GACC), (c) N‐malonyl‐ACC (MACC) and (d) jasmonyl‐ACC (JA‐ACC) content in flower, leaves and roots of the tomato cv UniDarkwin (UD) grafted onto MT (UD/WT), epi (high ethylene production, UD/*epi*) and *ACCD* (low ethylene production, UD/*ACCD*) rootstocks after 14 weeks growing under control and salt (75 mM NaCl) treatment. Bars indicate mean values ± SE. Different letters indicate significant differences among graft combinations (*n* = 6; *P* < 0.05) within and between treatments. Results of two‐way ANOVA (*P* values reported) for genotype (G), treatment (T) and their interaction (G × T) are indicated in the top right of the panel. *, ** and *** indicate statistically significant differences at *P* < 0.05, *P* < 0.01 and *P* < 0.001, respectively.

**Figure S3.** Global transcriptomic variation, gene clustering and functional enrichment analysis under salt stress. (a) Principal component analysis (PCA) of normalised RNA‐seq expression data. (b) Hierarchical clustering of expressed genes. Specific expression clusters are highlighted in blue, violet and green for UD/WT, UD/*epi* and UD/*ACCD* plants under salinity conditions, respectively. GO biological process enrichment analysis of the blue (c), violet (d) and green (e) clusters. (f) Network analysis of genes involved in sterol and steroid process (in cluster green).

**Figure S4.** Heatmap analysis of genes related to phosphate metabolism, sodium homeostasis and sterol biosynthesis in leaves under salinity. Heatmap representation of differentially expressed genes associated with (a) phosphate metabolism and transport, (b) sodium transport and homeostasis and (c) sterol biosynthesis in leaves of UD/WT, UD/*epi* and UD/*ACCD* tomato plants under control and salinity (75 mM NaCl) conditions. Gene expression values correspond to normalised RNA‐seq expression levels represented as Z‐score transformed counts per million (CPM). Red and blue colours indicate relative upregulation and downregulation, respectively.

**Figure S5.** Validation of gene expression by real‐time PCR quantification (qPCR) for *ACO1* (Solyc07g049530) (a), *ACO2* (Solyc12g005940) (b), *TAS14* (Solyc02g084850) (c), *PIP1.2* (Solyc01g094690) (d) and *CYP707A1* (Solyc08g005610) (e). qPCR data are normalised to housekeeping genes. Both qPCR and RNA‐seq values represent the relative expression of each gene compared to UD/WT under control conditions. Different letters indicate significant differences among graft combinations (*n* = 4; *P* < 0.05) assessing within and between treatments. Statistical comparisons were performed separately for qPCR and RNA‐seq.

**Figure S6.** Network analysis of genes and metabolites involved in ABA metabolism and signalling in the contrast C9 (UD/*ACCD* control versus salinity) in leaves, illustrating significant inferred interactions. The weight of the interactions between the different nodes from the network was calculated using the GENIE3 package in R. Nodes belonging the same network subset shared a consistent colour (ABA in yellow, ABA biosynthesis precursors in light blue, ABA catabolites in light green, and transcription factor, biosynthesis, catabolism, signalling and transport genes in red, dark blue, dark green, orange and purple, respectively). Significant interactions were defined as those whose weights ranked in the top 5% of the overall weight distribution. The thickness of the edges in the chart is based on the weight of the calculated edges.

**Table S1.** List of primers used for qRT‐PCR analysis.
**Table S2.** Two‐way‐ANOVA results (*P* value reported) for hormones and sugar levels, as shown in Figures 2 and 3A. *, ** and *** indicate statistically significant difference at *P* < 0.05, *P* < 0.01 and *P* < 0.001, respectively. G = Genotype; T = Treatment and GxT = Genotype × Treatment.
**Table S3.** Flavonoid analysis (cyanidin, genistein, luteolin, quercetin and taxifolin) in flowers, leaves and roots of the tomato cv UniDarkwin grafted onto MT (UD/WT), *epi* (high ethylene production, UD/*epi*) and *ACCD* (low ethylene production, UD/*ACCD*) rootstocks, expressed as peak area, after 14 weeks growing under control and salt (75 mM NaCl) treatment. Values represent peak area mean ± SE. Different letters indicate significant differences among graft combinations (*n* = 6; *P* < 0.05) within and between treatments. Results of two‐way ANOVA (*P* values reported) for genotype (G), treatment (T) and their interaction (G × T) are indicated on the right side of the Table. *, ** and *** indicate statistically significant difference at *P* < 0.05, *P* < 0.01 and *P* < 0.001, respectively.
**Table S4.** Amino acid concentrations polar neutrals (serine (Ser), asparagine (Asn), glutamine (Gln), tyrosine (Tyr)), nonpolar neutrals (valine (Val), leucine (Leu), isoleucine (Ile), proline (Pro), hydroxyproline (Hyp), phenylalanine (Phe), tryptophan (Trp)), acids (aspartic acid (Asp), glutamic acid (Glu)), bases (lysine (Lys), arginine (Arg), histidine (His)) in flowers of the tomato cv UniDarkwin grafted onto MT (UD/WT), *epi* (high ethylene production, UD/*epi*) and *ACCD* (low ethylene production, UD/*ACCD*) rootstocks, expressed as peak area, after 14 weeks growing under control and salt (75 mM NaCl) treatment. Values indicate mean peak area ± SE. Different letters indicate significant differences among graft combinations (*n* = 6; *P* < 0.05) within and between treatments. Results of two‐way ANOVA (*p* values reported) for genotype (G), treatment (T) and their interaction (G × T) are indicated on the right side of the Table. *, ** and *** indicate statistically significant difference at *P* < 0.05, *P* < 0.01 and *P* < 0.001, respectively.
**Table S5.** Library of expressed genes after filtering and normalisation. Expression levels are reported as counts per million (CPM).
**Table S6.** Ethylene‐related genes. Values represent mean relative expression (AveExpr) and adjusted *P* value (Adj.P.val). Colours indicate a gradient scale, with deep red denoting the highest expression values and deep blue the lowest expression values for each gene.
**Table S7.** Abscisic acid‐related genes. Values represent mean relative expression (AveExpr) and adjusted *P* value (Adj.P.val). Colours indicate a gradient scale, with deep red denoting the highest expression values and deep blue the lowest expression values for each gene.
**Table S8.** Gibberellin‐related genes. Values represent mean relative expression (AveExpr) and adjusted *P* value (Adj.P.val). Colours indicate a gradient scale, with deep red denoting the highest expression values and deep blue the lowest expression values for each gene.
**Table S9.** Cytokinin‐related genes. Values represent mean relative expression (AveExpr) and adjusted *P* value (Adj.P.val). Colours indicate a gradient scale, with deep red denoting the highest expression values and deep blue the lowest expression values for each gene.
**Table S10.** Jasmonic acid‐related genes. Values represent mean relative expression (AveExpr) and adjusted *P* value (Adj.P.val). Colours indicate a gradient scale, with deep red denoting the highest expression values and deep blue the lowest expression values for each gene.
**Table S11.** analysis of the Top 20 upregulated and top 20 downregulated genes used for network construction in the C4 Comparison (UD/*ACCD* salinity versus UD/WT salinity), depicting significant inferred interactions. The network was constructed using the GENIE3 package in R. The threshold for establishing edges between nodes was set to 0.1.
**Table S12.** Top 20 upregulated and top 20 downregulated genes for network construction in the C9 Comparison (UD/*ACCD* control versus salinity), depicting significant inferred interactions. The network was constructed using the GENIE3 package in R. The threshold for establishing edges between nodes was set to 0.1.
**Table S13.** Top 20 upregulated and top 20 downregulated genes for network construction in the C7 Comparison (UD/WT control versus salinity), depicting significant inferred interactions. The network was constructed using the GENIE3 package in R. The threshold for establishing edges between nodes was set to 0.1.
**Table S14.** Top 20 upregulated and top 20 downregulated genes for network construction in the C8 Comparison (UD/*epi* control versus salinity), depicting significant inferred interactions. The network was constructed using the GENIE3 package in R. The threshold for establishing edges between nodes was set to 0.1.
**Table S15.** Selected differentially expressed genes associated with phosphate metabolism, sodium homeostasis and sterol biosynthesis, including gene annotation, log2FC and adjusted P values.

## Data Availability

The RNA‐seq datasets generated and analysed during the current study are available in the NCBI Sequence Read Archive (SRA) under accession number PRJNA1347747.

## References

[tpj71133-bib-0001] Achard, P. , Cheng, H. , De Grauwe, L. , Decat, J. , Schoutteten, H. , Moritz, T. et al. (2006) Integration of plant responses to environmentally activated phytohormonal signals. Science (80‐), 311, 91–94. 10.1126/science.1118642 16400150

[tpj71133-bib-0002] Acosta‐Motos, J.R. , Ortuño, M.F. , Bernal‐Vicente, A. , Diaz‐Vivancos, P. , Sanchez‐Blanco, M.J. & Hernandez, J.A. (2017) Plant responses to Salt stress: adaptive mechanisms. Agronomy, 7, 18.

[tpj71133-bib-0003] Albacete, A. , Cantero‐Navarro, E. , Balibrea, M.E. , Großkinsky, D.K. , , la de Cruz González, M. , Martínez‐Andújar, C. et al. (2014) Hormonal and metabolic regulation of tomato fruit sink activity and yield under salinity. Journal of Experimental Botany, 65, 6081–6095. 10.1093/jxb/eru347 PMC4203140 25170099

[tpj71133-bib-0004] Albacete, A. , Ghanem, M.E. , Dodd, I.C. & Pérez‐Alfocea, F. (2010) Principal component analysis of hormone profiling data suggests an important role for cytokinins in regulating leaf growth and senescence of salinized tomato. Plant Signaling & Behavior, 5, 45–48. 10.4161/psb.5.1.10120 PMC2835956 20592807

[tpj71133-bib-0005] Albacete, A. , Ghanem, M.E. , Martínez‐Andújar, C. , Acosta, M. , Sánchez‐Bravo, J. , Martínez, V. et al. (2008) Hormonal changes in relation to biomass partitioning and shoot growth impairment in salinized tomato (*Solanum lycopersicum* L.) plants. Journal of Experimental Botany, 59, 4119–4131. 10.1093/jxb/ern251 PMC2639025 19036841

[tpj71133-bib-0006] Albacete, A. , Martínez‐Andújar, C. , Ghanem, M.E. , Acosta, M. , Sánchez‐Bravo, J. , Asins, M.J. et al. (2009) Rootstock‐mediated changes in xylem ionic and hormonal status are correlated with delayed leaf senescence, and increased leaf area and crop productivity in salinized tomato. Plant, Cell & Environment, 32, 928–938. 10.1111/j.1365-3040.2009.01973.x 19302168

[tpj71133-bib-0007] Albacete, A. , Martínez‐Andújar, C. , Martínez‐Pérez, A. , Thompson, A.J. , Dodd, I.C. & Pérez‐Alfocea, F. (2015) Unravelling rootstock× scion interactions to improve food security. Journal of Experimental Botany, 66, 2211–2226. 10.1093/jxb/erv027 PMC4986720 25754404

[tpj71133-bib-0008] Albacete, A. , Martínez‐Andújar, C. & Pérez‐Alfocea, F. (2014) Hormonal and metabolic regulation of source–sink relations under salinity and drought: from plant survival to crop yield stability. Biotechnology Advances, 32, 12–30. 10.1016/j.biotechadv.2013.10.005 24513173

[tpj71133-bib-0009] Álvarez‐Hernández, M.H. , Martínez‐Hernández, G.B. , Avalos‐Belmontes, F. , Miranda‐Molina, F.D. & Artés‐Hernández, F. (2020) Postharvest quality retention of apricots by using a novel sepiolite–loaded potassium permanganate ethylene scavenger. Postharvest Biology and Technology, 160, 111061.

[tpj71133-bib-0010] Argyros, R.D. , Mathews, D.E. , Chiang, Y.‐H. , Palmer, C.M. , Thibault, D.M. , Etheridge, N. et al. (2008) Type B response regulators of Arabidopsis play key roles in cytokinin signaling and plant development. Plant Cell, 20, 2102–2116. 10.1105/tpc.108.059584 PMC2553617 18723577

[tpj71133-bib-0011] Bai, Q. , Huang, Y. & Shen, Y. (2021) The physiological and molecular mechanism of abscisic acid in regulation of fleshy fruit ripening. Frontiers in Plant Science, 11. https://www.frontiersin.org/articles/10.3389/fpls.2020.619953, 619953. 10.3389/fpls.2020.619953 PMC7829184 33505417

[tpj71133-bib-0012] de Bang, T.C. , Husted, S. , Laursen, K.H. , Persson, D.P. & Schjoerring, J.K. (2021) The molecular–physiological functions of mineral macronutrients and their consequences for deficiency symptoms in plants. New Phytologist, 229, 2446–2469. 10.1111/nph.17074 33175410

[tpj71133-bib-0013] Barry, C.S. , Fox, E.A. , Yen, H. , Lee, S. , Ying, T. , Grierson, D. et al. (2001) Analysis of the ethylene response in the epinastic mutant of tomato. Plant Physiology, 127, 58–66. 10.1104/pp.127.1.58 PMC117962 11553734

[tpj71133-bib-0014] Belimov, A.A. , Ulianich, P.S. , Syrova, D.S. , Shaposhnikov, A.I. , Safronova, V.I. & Dodd, I.C. (2022) Modulation of tomato root architecture and root hair traits by *Pseudomonas brassicacearum* and *Variovorax paradoxus* containing 1‐aminocyclopropane‐1‐carboxylate deaminase. Biologia Plantarum, 66, 228–239.

[tpj71133-bib-0015] Bhadouria, J. & Giri, J. (2022) Purple acid phosphatases: roles in phosphate utilization and new emerging functions. Plant Cell Reports, 41, 33–51. Available from: 10.1007/s00299-021-02773-7 34402946

[tpj71133-bib-0016] Bhardwaj, S. , Sharma, D. , Jan, S. , Singh, R. , Bhardwaj, R. & Kapoor, D. (2022) Crosstalk of ethylene and other phytohormones in the regulation of plant development. In: Ethylene in Plant Biology. Hoboken, NJ, USA: Wiley, pp. 17–31. Available from: 10.1002/9781119744719.ch2

[tpj71133-bib-0017] Bhise, K.K. & Dandge, P.B. (2019) Mitigation of salinity stress in plants using plant growth promoting bacteria. Symbiosis, 79, 191–204.

[tpj71133-bib-0018] Bisht, D. , Mishra, S. , Bihani, S.C. , Seth, T. , Srivastava, A.K. & Pandey, G.K. (2025) Salt stress tolerance and calcium signalling components: where we stand and how far we can go? Journal of Plant Growth Regulation, 44, 1429–1447.

[tpj71133-bib-0019] Bletsos, F.A. & Olympios, C.M. (2008) Rootstocks and grafting of tomatoes, peppers and eggplants for soil‐borne disease resistance, improved yield and quality. European Journal of Plant Science and Biotechnology, 2, 62–73.

[tpj71133-bib-0020] Borghi, M. & Fernie, A.R. (2017) Floral metabolism of sugars and amino acids: implications for pollinators' preferences and seed and fruit set. Plant Physiology, 175, 1510–1524. Available from: 10.1104/pp.17.01164 PMC5717749 28986424

[tpj71133-bib-0021] Burgess, D.J. (2017) Branching out for crop improvement. Nature Reviews. Genetics, 18, 393. Available from: 10.1038/nrg.2017.48 28579607

[tpj71133-bib-0022] Cao, Y.‐R. , Chen, S.‐Y. & Zhang, J.‐S. (2008) Ethylene signaling regulates salt stress response: an overview. Plant Signaling & Behavior, 3, 761–763. 10.4161/psb.3.10.5934 PMC2634369 19513226

[tpj71133-bib-0023] Carvalho, R.F. , Campos, M.L. , Pino, L.E. , Crestana, S.L. , Zsögön, A. , Lima, J.E. et al. (2011) Convergence of developmental mutants into a single tomato model system: ‘Micro‐Tom’ as an effective toolkit for plant development research. Plant Methods, 7, 18. 10.1186/1746-4811-7-18 PMC3146949 21714900

[tpj71133-bib-0024] Chapin, F.S., III & Wardlaw, I.F. (1988) Effect of phosphorus deficiency on source‐sink interactions between the flag leaf and developing grain in barley. Journal of Experimental Botany, 39, 165–177.

[tpj71133-bib-0025] Chen, H. , Bullock, D.A., Jr. , Alonso, J.M. & Stepanova, A.N. (2021) To fight or to grow: the balancing role of ethylene in plant abiotic stress responses. Plants, 11(1), 33. 10.3390/plants11010033 PMC8747122 35009037

[tpj71133-bib-0026] Cohen, R. , Edelstein, M. & Aktas, H. (2010) The use of plant grafting and plant growth regulators for enhancing abiotic stress tolerance in vegetable transplants. Scientia Horticulturae, 127, 119–126.

[tpj71133-bib-0027] Ditta, G. , Pinyopich, A. , Robles, P. , Pelaz, S. & Yanofsky, M.F. (2004) The SEP4 gene of Arabidopsis thaliana functions in floral organ and meristem identity. Current Biology, 14, 1935–1940. 10.1016/j.cub.2004.10.028 15530395

[tpj71133-bib-0028] Dodd, I.C. (2005) Root‐to‐shoot signalling: assessing the roles of ‘up’ in the up and down world of long‐distance signalling in planta. Plant and Soil, 274, 251–270.

[tpj71133-bib-0029] Duan, L. , Dietrich, D. , Ng, C.H. , Chan, P.M.Y. , Bhalerao, R. , Bennett, M.J. et al. (2013) Endodermal ABA signaling promotes lateral root quiescence during salt stress in Arabidopsis seedlings. Plant Cell, 25, 324–341. 10.1105/tpc.112.107227 PMC3584545 23341337

[tpj71133-bib-0030] FAOSTAT . (2023) FAO Statical Database. https://www.fao.org/faostat/. Accessed October 10, 2025.

[tpj71133-bib-0031] Fernández‐García, N. , Olmos, E. , Bardisi, E. , García‐De la Garma, J. , López‐Berenguer, C. & Rubio‐Asensio, J.S. (2014) Intrinsic water use efficiency controls the adaptation to high salinity in a semi‐arid adapted plant, henna (*Lawsonia inermis* L.). Journal of Plant Physiology, 171, 64–75. 10.1016/j.jplph.2013.11.004 24484959

[tpj71133-bib-0032] Fujino, D.W. , Burger, D.W. , Yang, S.F. & Bradford, K.J. (1988) Characterization of an ethylene overproducing mutant of tomato (*Lycopersicon esculentum* Mill. cultivar VFN8). Plant Physiology, 88, 774–779. 10.1104/pp.88.3.774 PMC1055659 16666382

[tpj71133-bib-0033] Ge, S.X. , Jung, D. & Yao, R. (2020) ShinyGO: a graphical gene‐set enrichment tool for animals and plants. Bioinformatics, 36, 2628–2629. 10.1093/bioinformatics/btz931 PMC7178415 31882993

[tpj71133-bib-0034] Ge, S.X. , Son, E.W. & Yao, R. (2018) iDEP: an integrated web application for differential expression and pathway analysis of RNA‐Seq data. BMC Bioinformatics, 19, 1–24. 10.1186/s12859-018-2486-6 PMC6299935 30567491

[tpj71133-bib-0035] Geissler, N. , Hussin, S. & Koyro, H.‐W. (2009) Elevated atmospheric CO_2_ concentration ameliorates effects of NaCl salinity on photosynthesis and leaf structure of *Aster tripolium* L. Journal of Experimental Botany, 60, 137–151. 10.1093/jxb/ern271 PMC3071763 19036838

[tpj71133-bib-0036] Ghanem, M.E. , Albacete, A. , Martínez‐Andújar, C. , Acosta, M. , Romero‐Aranda, R. , Dodd, I.C. et al. (2008) Hormonal changes during salinity‐induced leaf senescence in tomato (*Solanum lycopersicum* L.). Journal of Experimental Botany, 59, 3039–3050. 10.1093/jxb/ern153 PMC2504355 18573798

[tpj71133-bib-0037] Ghanem, M.E. , Albacete, A. , Smigocki, A.C. , Frébort, I. , Pospíšilová, H. , Martínez‐Andújar, C. et al. (2011) Root‐synthesized cytokinins improve shoot growth and fruit yield in salinized tomato (*Solanum lycopersicum* L.) plants. Journal of Experimental Botany, 62, 125–140. 10.1093/jxb/erq266 PMC2993914 20959628

[tpj71133-bib-0038] Göttlinger, T. & Lohaus, G. (2023) Origin and function of amino acids in nectar and nectaries of Pitcairnia species with particular emphasis on alanine and glutamine. Plants, 13, 23. 10.3390/plants13010023 PMC10780904 38202331

[tpj71133-bib-0039] Grenzi, M. , Buratti, S. , Parmagnani, A.S. , Abdel Aziz, I. , Bernacka‐Wojcik, I. , Resentini, F. et al. (2023) Long‐distance turgor pressure changes induce local activation of plant glutamate receptor‐like channels. Current Biology, 33, 1019–1035. 10.1016/j.cub.2023.01.042 36796359

[tpj71133-bib-0040] Guida‐Johnson, B. , Abraham, E.M. & Cony, M.A. (2017) Soil salinization in irrigated drylands: prospects for restoration in Cuyo, Argentina. Revista de la Facultad de Ciencias Agrarias, Universidad Nacional de Cuyo, 49, 205–215.

[tpj71133-bib-0041] Guo, Q. , Liu, L. & Barkla, B.J. (2019) Membrane lipid remodeling in response to salinity. International Journal of Molecular Sciences, 20, 4264. 10.3390/ijms20174264 PMC6747501 31480391

[tpj71133-bib-0042] Hallmark, H.T. , Černý, M. , Brzobohatý, B. & Rashotte, A.M. (2020) Trans‐Zeatin‐N‐glucosides have biological activity in *Arabidopsis thaliana* . PLoS One, 15, e0232762. Available from: 10.1371/journal.pone.0232762 PMC7205299 32379789

[tpj71133-bib-0043] Hanchi, M. , Thibaud, M.C. , Légeret, B. , Kuwata, K. , Pochon, N. , Beisson, F. et al. (2018) The phosphate fast‐responsive genes *PECP1* and *PPsPase1* affect phosphocholine and phosphoethanolamine content. Plant Physiology, 176, 2943–2962. 10.1104/pp.17.01246 PMC5884592 29475899

[tpj71133-bib-0044] Hosmani, P.S. , Flores‐Gonzalez, M. , van de Geest, H. , Hosmani, P.S. , Flores‐Gonzalez, M. , van de Geest, H. et al. (2019) An improved de novo assembly and annotation of the tomato reference genome using single‐molecule sequencing, Hi‐C proximity ligation and optical maps. *bioRxiv: the preprint server for biology*, 767764.

[tpj71133-bib-0045] Houben, M. , Vaughan‐Hirsch, J. , Pattyn, J. , Mou, W. , Roden, S. , Roig Martinez, A. et al. (2026) 1‐Aminocyclopropane‐1‐carboxylic acid oxidase determines the fate of ethylene biosynthesis in a tissue‐specific way. Plant Physiology, 200, kiag025. 10.1093/plphys/kiag025 41604457

[tpj71133-bib-0046] Iqbal, N. , Masood, A. & Khan, N.A. (2012) Phytohormones in salinity tolerance: ethylene and gibberellins cross talk. In: Phytohormones and abiotic stress tolerance in plants. Berlin, Heidelberg, Germany: Springer, pp. 77–98. Available from: https://link.springer.com/chapter/10.1007/978-3-642-25829-9_3

[tpj71133-bib-0047] Ishida, K. , Yamashino, T. , Yokoyama, A. & Mizuno, T. (2008) Three type‐B response regulators, *ARR1*, *ARR10* and *ARR12*, play essential but redundant roles in cytokinin signal transduction throughout the life cycle of *Arabidopsis thaliana* . Plant & Cell Physiology, 49, 47–57. 10.1093/pcp/pcm165 18037673

[tpj71133-bib-0048] Jeon, J. , Kim, N.Y. , Kim, S. , Kang, N.Y. , Novák, O. , Ku, S.J. et al. (2010) A subset of cytokinin two‐component signaling system plays a role in cold temperature stress response in Arabidopsis. The Journal of Biological Chemistry, 285, 23371–23386. 10.1074/jbc.M109.096644 PMC2906329 20463025

[tpj71133-bib-0049] Jetha, K. , Theißen, G. & Melzer, R. (2014) Arabidopsis SEPALLATA proteins differ in cooperative DNA‐binding during the formation of floral quartet‐like complexes. Nucleic Acids Research, 42, 10927–10942. 10.1093/nar/gku755 PMC4176161 25183521

[tpj71133-bib-0050] Julkowska, M.M. , Koevoets, I.T. , Mol, S. , Hoefsloot, H. , Feron, R. , Tester, M.A. et al. (2017) Genetic components of root architecture remodeling in response to salt stress. Plant Cell, 29, 3198–3213. 10.1105/tpc.16.00680 PMC5757256 29114015

[tpj71133-bib-0051] Jung, H. , Ali, S. , Kim, J.Y. & Kim, W.‐C. (2018) Transgenic Arabidopsis expressing acdS gene of *Pseudomonas veronii‐KJ* alleviate the adverse effects of salt and water‐logging stress. Plant Breeding and Biotechnology, 6, 221–232.

[tpj71133-bib-0052] Jung, J.‐Y. , Shin, R. & Schachtman, D.P. (2009) Ethylene mediates response and tolerance to potassium deprivation in Arabidopsis. Plant Cell, 21, 607–621. 10.1105/tpc.108.063099 PMC2660615 19190240

[tpj71133-bib-0053] Kahlaoui, B. , Hachicha, M. , Misle, E. , Fidalgo, F. & Teixeira, J. (2018) Physiological and biochemical responses to the exogenous application of proline of tomato plants irrigated with saline water. Journal of the Saudi Society of Agricultural Sciences, 17, 17–23. https://www.sciencedirect.com/science/article/pii/S1658077X1500048X

[tpj71133-bib-0054] Kawa, D. , Meyer, A.J. , Dekker, H.L. , Abd‐el‐Haliem, A.M. , Gevaert, K. , van de Slijke, E. et al. (2020) SnRK2 protein kinases and mRNA decapping machinery control root development and response to salt. Plant Physiology, 182, 361–377. 10.1104/pp.19.00818 PMC6945840 31570508

[tpj71133-bib-0055] Keshishian, E. (2018) CRF2 and its role in cytokinin response and abiotic stress.

[tpj71133-bib-0056] Klee, H.J. , Hayford, M.B. , Kretzmer, K.A. , Barry, G.F. & Kishore, G.M. (1991) Control of ethylene synthesis by expression of a bacterial enzyme in transgenic tomato plants. Plant Cell, 3, 1187–1193. 10.1105/tpc.3.11.1187 PMC160085 1821764

[tpj71133-bib-0057] Kumar, M.S.S. , Ali, K. , Dahuja, A. & Tyagi, A. (2015) Role of phytosterols in drought stress tolerance in rice. Plant Physiology and Biochemistry, 96, 83–89. 10.1016/j.plaphy.2015.07.014 26233709

[tpj71133-bib-0058] Kushiro, T. , Okamoto, M. , Nakabayashi, K. , Yamagishi, K. , Kitamura, S. , Asami, T. et al. (2004) The Arabidopsis cytochrome P450 *CYP707A* encodes ABA 8′‐hydroxylases: key enzymes in ABA catabolism. The EMBO Journal, 23, 1647–1656. 10.1038/sj.emboj.7600121 PMC391058 15044947

[tpj71133-bib-0059] Larriba, E. , Sánchez‐García, A.B. , Martínez‐Andújar, C. , Albacete, A. & Pérez‐Pérez, J.M. (2021) Tissue‐specific metabolic reprogramming during wound‐induced organ formation in tomato hypocotyl explants. International Journal of Molecular Sciences, 22, 10112. 10.3390/ijms221810112 PMC8466849 34576275

[tpj71133-bib-0060] Lechner, M. , Findeiß, S. , Steiner, L. , Marz, M. , Stadler, P.F. & Prohaska, S.J. (2011) Proteinortho: detection of (co‐) orthologs in large‐scale analysis. BMC Bioinformatics, 12, 1–9. 10.1186/1471-2105-12-124 PMC3114741 21526987

[tpj71133-bib-0061] Lewis, D.R. , Negi, S. , Sukumar, P. & Muday, G.K. (2011) Ethylene inhibits lateral root development, increases IAA transport and expression of *PIN3* and *PIN7* auxin efflux carriers. Development, 138, 3485–3495. 10.1242/dev.065102 21771812

[tpj71133-bib-0062] Li, C. , Liu, X. , Ruan, H. , Zhang, J. , Xie, F. , Gai, J. et al. (2020) *GmWRKY45* enhances tolerance to phosphate starvation and Salt stress, and changes fertility in transgenic Arabidopsis. Frontiers in Plant Science, 10. https://www.frontiersin.org/articles/10.3389/fpls.2019.01714, 1714. 10.3389/fpls.2019.01714 PMC7000756 32082335

[tpj71133-bib-0063] Li, Y. , Liu, F. , Li, P. , Wang, T. , Zheng, C. & Hou, B. (2020) An Arabidopsis cytokinin‐modifying glycosyltransferase *UGT76C2* improves drought and salt tolerance in rice. Frontiers in Plant Science, 11, 560696. 10.3389/fpls.2020.560696 PMC7674613 33224159

[tpj71133-bib-0064] Liao, Y. , Smyth, G.K. & Shi, W. (2019) The R package Rsubread is easier, faster, cheaper and better for alignment and quantification of RNA sequencing reads. Nucleic Acids Research, 47, e47. 10.1093/nar/gkz114 PMC6486549 30783653

[tpj71133-bib-0065] Liu, L. , Wang, X. & Chang, C. (2022) Toward a smart skin: harnessing cuticle biosynthesis for crop adaptation to drought, salinity, temperature, and ultraviolet stress. Frontiers in Plant Science, 13. Available from: 10.3389/fpls.2022.961829 PMC9358614 35958191

[tpj71133-bib-0066] Liu, X. , Zeng, Y. , Hasan, M.M. , Ghimire, S. , Jiang, H. , Qi, S. et al. (2024) Diverse functional interactions between ABA and ethylene in plant development and responses to stress. Physiologia Plantarum, 176, e70000. 10.1111/ppl.70000 39686889

[tpj71133-bib-0067] Liu, Y. , Zhang, M. , Meng, Z. , Wang, B. & Chen, M. (2020) Research progress on the roles of cytokinin in plant response to stress. International Journal of Molecular Sciences, 21, 6574. 10.3390/ijms21186574 PMC7555750 32911801

[tpj71133-bib-0068] del Mar Parra, M. , del Pozo, O. , Luna, R. , Godoy, J.A. & Pintor‐Toro, J.A. (1996) Structure of the dehydrin tas 14 gene of tomato and its developmental and environmental regulation in transgenic tobacco. Plant Molecular Biology, 32, 453–460. 10.1007/BF00019097 8980494

[tpj71133-bib-0069] Martínez‐Andújar, C. , Albacete, A. , Martínez‐Pérez, A. , Pérez‐Pérez, J.M. , Asins, M.J. & Pérez‐Alfocea, F. (2016) Root‐to‐shoot hormonal communication in contrasting rootstocks suggests an important role for the ethylene precursor aminocyclopropane‐1‐carboxylic acid in mediating plant growth under low‐potassium nutrition in tomato. Frontiers in Plant Science, 7, 1782. 10.3389/fpls.2016.01782 PMC5126091 27965690

[tpj71133-bib-0070] Martínez‐Andújar, C. , Albacete, A. & Pérez‐Alfocea, F. (2018) Rootstocks for increasing yield stability and sustainability in vegetable crops. *XXX International Horticultural Congress IHC2018: II International Symposium on Soilless Culture and VIII International, 1273, 449–470*.

[tpj71133-bib-0071] Martínez‐Andújar, C. , Martínez‐Pérez, A. , Albacete, A. , Martínez‐Melgarejo, P.A. , Dodd, I.C. , Thompson, A.J. et al. (2021) Overproduction of ABA in rootstocks alleviates salinity stress in tomato shoots. Plant, Cell & Environment, 44, 2966–2986. Available from: 10.1111/pce.14121 34053093

[tpj71133-bib-0072] Martínez‐Andújar, C. , Martínez‐Pérez, A. , Ferrández‐Ayela, A. , Albacete, A. , Martínez‐Melgarejo, P.A. , Dodd, I.C. et al. (2020) Impact of overexpression of 9‐cis‐epoxycarotenoid dioxygenase on growth and gene expression under salinity stress. Plant Science, 295, 110268. 10.1016/j.plantsci.2019.110268 32534608

[tpj71133-bib-0073] Martínez‐Andújar, C. , Ruiz‐Lozano, J.M. , Dodd, I.C. , Albacete, A. & Pérez‐Alfocea, F. (2017) Hormonal and nutritional features in contrasting rootstock‐mediated tomato growth under low‐phosphorus nutrition. Frontiers in Plant Science, 8, 533. 10.3389/fpls.2017.00533 PMC5386964 28443121

[tpj71133-bib-0074] Martínez‐Andújar, C. , Youssef, R.B. , Prudencio, Á.S. , Ormazabal, M. , Martín‐Rodríguez, J.Á. , Albacete, A. et al. (2023) Bumblebees sense rootstock‐mediated nutrition and fertilization regime in tomato. Plant and Soil, 486, 293–306. Available from: 10.1007/s11104-023-05868-0

[tpj71133-bib-0075] Martínez‐Melgarejo, P.A. (2022) Role of ABA in the regulation of root architecture under abiotic stress conditions in tomato (*Solanum lycopersicum*) and soybean (*Glycine max* (L.) Merr.). Murcia, Spain: University of Murcia. Available from: https://dialnet.unirioja.es/servlet/tesis?codigo=303267

[tpj71133-bib-0076] Mason, M.G. , Jha, D. , Salt, D.E. , Tester, M. , Hill, K. , Kieber, J.J. et al. (2010) Type‐B response regulators ARR1 and ARR12 regulate expression of AtHKT1; 1 and accumulation of sodium in Arabidopsis shoots. The Plant Journal, 64, 753–763. 10.1111/j.1365-313X.2010.04366.x 21105923

[tpj71133-bib-0078] Mou, W. , Kao, Y.‐T. , Michard, E. , Simon, A.A. , Li, D. , Wudick, M.M. et al. (2020) Ethylene‐independent signaling by the ethylene precursor ACC in Arabidopsis ovular pollen tube attraction. Nature Communications, 11, 4082. Available from: 10.1038/s41467-020-17819-9 PMC7429864 32796832

[tpj71133-bib-0079] Mou, W. , Khare, R. , Polko, J.K. , Taylor, I. , Xu, J. , Xue, D. et al. (2025) Ethylene‐independent modulation of root development by ACC via downregulation of WOX5 and group I CLE peptide expression. Proceedings of the National Academy of Sciences, 122, e2417735122. 10.1073/pnas.2417735122 PMC11831204 39908106

[tpj71133-bib-0080] Munns, R. (2002) Salinity, growth and phytohormones. In: Salinity: environment‐plants‐molecules. Springer, pp. 271–290. https://link.springer.com/book/10.1007/0‐306‐48155‐3#about‐this‐book

[tpj71133-bib-0081] Munns, R. & Tester, M. (2008) Mechanisms of salinity tolerance. Annual Review of Plant Biology, 59, 651–681. 10.1146/annurev.arplant.59.032607.092911 18444910

[tpj71133-bib-0082] Murray, A.F. , Leonhardt, S.D. , Stout, J.C. , Ruedenauer, F.A. , Vanderplanck, M. & Russo, L. (2025) Pollen‐derived fatty acids and amino acids mediate variance in pollinator visitation. Journal of Chemical Ecology, 51, 7. Available from: 10.1007/s10886-025-01552-y 39853498

[tpj71133-bib-0083] Naing, A.H. , Maung, T. & Kim, C.K. (2021) The ACC deaminase‐producing plant growth‐promoting bacteria: influences of bacterial strains and ACC deaminase activities in plant tolerance to abiotic stress. Physiologia Plantarum, 173, 1992–2012. 10.1111/ppl.13545 34487352

[tpj71133-bib-0084] Nakabayashi, R. , Yonekura‐Sakakibara, K. , Urano, K. , Suzuki, M. , Yamada, Y. , Nishizawa, T. et al. (2014) Enhancement of oxidative and drought tolerance in Arabidopsis by overaccumulation of antioxidant flavonoids. The Plant Journal, 77, 367–379. 10.1111/tpj.12388 PMC4282528 24274116

[tpj71133-bib-0085] Nguyen, H.N. , Nguyen, T.Q. , Kisiala, A.B. & Emery, R.J.N. (2021) Beyond transport: cytokinin ribosides are translocated and active in regulating the development and environmental responses of plants. Planta, 254, 45. Available from: 10.1007/s00425-021-03693-2 34365553

[tpj71133-bib-0086] Nicolás, E. , Maestre‐Valero, J.F. , Alarcón, J.J. , Pedrero, F. , Vicente‐Sánchez, J. , Bernabé, A. et al. (2015) Effectiveness and persistence of arbuscular mycorrhizal fungi on the physiology, nutrient uptake and yield of Crimson seedless grapevine. The Journal of Agricultural Science, 153, 1084–1096.

[tpj71133-bib-0087] Nie, R. , Chen, D. , Hu, T. , Zhang, S. & Qu, G. (2025) A review: the role of Jasmonic acid in tomato flower and fruit development. Plant Molecular Biology Reporter, 43, 474–483.

[tpj71133-bib-0088] Ormazabal, M. , Prudencio, Á.S. , Martínez‐Melgarejo, P.A. , Martín‐Rodríguez, J.Á. , Ruiz‐Pérez, L. , Martínez‐Andújar, C. et al. (2024) Rootstock effects on tomato fruit composition and pollinator preferences in tomato. Horticulturae, 10, 992.

[tpj71133-bib-0089] Pan, Y. , Bradley, G. , Pyke, K. , Ball, G. , Lu, C. , Fray, R. et al. (2013) Network inference analysis identifies an APRR2‐like gene linked to pigment accumulation in tomato and pepper fruits. Plant Physiology, 161, 1476–1485. 10.1104/pp.112.212654 PMC3585610 23292788

[tpj71133-bib-0090] Park, C.H. , Roh, J. , Youn, J.‐H. , Son, S.‐H. , Park, J.H. , Kim, S.Y. et al. (2018) Arabidopsis ACC oxidase 1 coordinated by multiple signals mediates ethylene biosynthesis and is involved in root development. Molecules and Cells, 41, 923–932. https://www.sciencedirect.com/science/article/pii/S1016847823005629 10.14348/molcells.2018.0092 PMC6199567 30352493

[tpj71133-bib-0091] Peng, J. , Li, Z. , Wen, X. , Li, W. , Shi, H. , Yang, L. et al. (2014) Salt‐induced stabilization of *EIN3/EIL1* confers salinity tolerance by deterring ROS accumulation in Arabidopsis. PLoS Genetics, 10, e1004664. 10.1371/journal.pgen.1004664 PMC4199496 25330213

[tpj71133-bib-0092] Pérez‐Alfocea, F. , Albacete, A. , Ghanem, M.E. & Dodd, I.C. (2010) Hormonal regulation of source–sink relations to maintain crop productivity under salinity: a case study of root‐to‐shoot signalling in tomato. Functional Plant Biology, 37, 592–603.

[tpj71133-bib-0093] Pérez‐Alfocea, F. , Yeboah, S. & Dodd, I.C. (2021) Rootstocks to improve root function and resource‐use efficiency. In: Understanding and improving crop root function. Burleigh Dodds Science Publishing, pp. 561–588. Available from: 10.1201/9781003048015

[tpj71133-bib-0094] Pfaffl, M.W. (2001) A new mathematical model for relative quantification in real‐time RT–PCR. Nucleic Acids Research, 29, e45. 10.1093/nar/29.9.e45 PMC55695 11328886

[tpj71133-bib-0095] Pierik, R. , Tholen, D. , Poorter, H. , Visser, E.J.W. & Voesenek, L.A.C.J. (2006) The Janus face of ethylene: growth inhibition and stimulation. Trends in Plant Science, 11, 176–183. 10.1016/j.tplants.2006.02.006 16531097

[tpj71133-bib-0096] Qin, L. , Wang, L. , Guo, Y. , Li, Y. , Ümüt, H. & Wang, Y. (2017) An ERF transcription factor from *Tamarix hispida*, *ThCRF1*, can adjust osmotic potential and reactive oxygen species scavenging capability to improve salt tolerance. Plant Science, 265, 154–166. 10.1016/j.plantsci.2017.10.006 29223337

[tpj71133-bib-0097] Rehman, A. , Azhar, M.T. , Hinze, L. , Qayyum, A. , Li, H. , Peng, Z. et al. (2021) Insight into abscisic acid perception and signaling to increase plant tolerance to abiotic stress. Journal of Plant Interactions, 16, 222–237. Available from: 10.1080/17429145.2021.1925759

[tpj71133-bib-0098] Reuscher, S. , Akiyama, M. , Mori, C. , Aoki, K. , Shibata, D. & Shiratake, K. (2013) Genome‐wide identification and expression analysis of aquaporins in tomato. PLoS One, 8, e79052. 10.1371/journal.pone.0079052 PMC3834038 24260152

[tpj71133-bib-0099] Riyazuddin, R. , Verma, R. , Singh, K. , Nisha, N. , Keisham, M. , Bhati, K.K. et al. (2020) Ethylene: a master regulator of salinity stress tolerance in plants. Biomolecules, 10, 959. 10.3390/biom10060959 PMC7355584 32630474

[tpj71133-bib-0100] Rogowska, A. & Szakiel, A. (2020) The role of sterols in plant response to abiotic stress. Phytochemistry Reviews, 19, 1525–1538.

[tpj71133-bib-0101] Savvas, D. , Savva, A. , Ntatsi, G. , Ropokis, A. , Karapanos, I. , Krumbein, A. et al. (2011) Effects of three commercial rootstocks on mineral nutrition, fruit yield, and quality of salinized tomato. Journal of Plant Nutrition and Soil Science, 174, 154–162.

[tpj71133-bib-0102] Shahzad, K. , Hussain, S. , Arfan, M. , Hussain, S. , Waraich, E.A. , Zamir, S. et al. (2021) Exogenously applied gibberellic acid enhances growth and salinity stress tolerance of maize through modulating the morpho‐physiological, biochemical and molecular attributes. Biomolecules, 11, 1005. 10.3390/biom11071005 PMC8301807 34356629

[tpj71133-bib-0103] Shi, Y. , Tian, S. , Hou, L. , Huang, X. , Zhang, X. , Guo, H. et al. (2012) Ethylene signaling negatively regulates freezing tolerance by repressing expression of *CBF* and type‐a *ARR* genes in Arabidopsis. Plant Cell, 24, 2578–2595. 10.1105/tpc.112.098640 PMC3406918 22706288

[tpj71133-bib-0104] Skirycz, A. , Vandenbroucke, K. , Clauw, P. , Maleux, K. , de Meyer, B. , Dhondt, S. et al. (2011) Survival and growth of Arabidopsis plants given limited water are not equal. Nature Biotechnology, 29, 212–214. 10.1038/nbt.1800 21390020

[tpj71133-bib-0105] Šmehilová, M. , Dobrůšková, J. , Novák, O. , Takáč, T. & Galuszka, P. (2016) Cytokinin‐specific glycosyltransferases possess different roles in cytokinin homeostasis maintenance. Frontiers in Plant Science, 7, 1264 https://www.frontiersin.org/articles/10.3389/fpls.2016.01264 10.3389/fpls.2016.01264 PMC4993776 27602043

[tpj71133-bib-0106] Staswick, P.E. & Tiryaki, I. (2004) The oxylipin signal jasmonic acid is activated by an enzyme that conjugates it to isoleucine in Arabidopsis. Plant Cell, 16, 2117–2127. 10.1105/tpc.104.023549 PMC519202 15258265

[tpj71133-bib-0107] Su, R. , Zhang, Z. , Chang, C. , Peng, Q. , Cheng, X. , Pang, J. et al. (2022) Interactive effects of phosphorus fertilization and salinity on plant growth, phosphorus and sodium status, and tartrate exudation by roots of two alfalfa cultivars. Annals of Botany, 129, 53–64. 10.1093/aob/mcab124 PMC8752396 34582551

[tpj71133-bib-0108] Sun, L. , Zhang, Q. , Wu, J. , Zhang, L. , Jiao, X. , Zhang, S. et al. (2014) Two rice authentic histidine phosphotransfer proteins, OsAHP1 and OsAHP2, mediate cytokinin signaling and stress responses in rice. Plant Physiology, 165, 335–345. 10.1104/pp.113.232629 PMC4012592 24578505

[tpj71133-bib-0109] Tao, J.‐J. , Chen, H.‐W. , Ma, B. , Zhang, W.‐K. , Chen, S.‐Y. & Zhang, J.‐S. (2015) The role of ethylene in plants under salinity stress. Frontiers in Plant Science, 6, 1059. 10.3389/fpls.2015.01059 PMC4661241 26640476

[tpj71133-bib-0110] Thalmann, M. & Santelia, D. (2017) Starch as a determinant of plant fitness under abiotic stress. The New Phytologist, 214, 943–951. Available from: 10.1111/nph.14491 28277621

[tpj71133-bib-0111] Tsang, D.L. , Edmond, C. , Harrington, J.L. & Nühse, T.S. (2011) Cell wall integrity controls root elongation via a general 1‐aminocyclopropane‐1‐carboxylic acid‐dependent, ethylene‐independent pathway. Plant Physiology, 156, 596–604. 10.1104/pp.111.175372 PMC3177261 21508182

[tpj71133-bib-0112] Vanderstraeten, L. , Depaepe, T. , Bertrand, S. & Van Der Straeten, D. (2019) The ethylene precursor ACC affects early vegetative development independently of ethylene signaling. Frontiers in Plant Science, 10, 1591. 10.3389/fpls.2019.01591 PMC6908520 31867034

[tpj71133-bib-0113] Vanderstraeten, L. & Van Der Straeten, D. (2017) Accumulation and transport of 1‐aminocyclopropane‐1‐carboxylic acid (ACC) in plants: current status, considerations for future research and agronomic applications. Frontiers in Plant Science, 8, 38. 10.3389/fpls.2017.00038 PMC5258695 28174583

[tpj71133-bib-0114] Velmurugan, A. , Swarnam, P. , Subramani, T. , Meena, B. & Kaledhonkar, M.J. (2020) Water demand and salinity. *Desalination‐challenges Oppor* .

[tpj71133-bib-0115] Wang, Y. , Liu, C. , Li, K. , Sun, F. , Hu, H. , Li, X. et al. (2007) Arabidopsis *EIN2* modulates stress response through abscisic acid response pathway. Plant Molecular Biology, 64, 633–644. 10.1007/s11103-007-9182-7 17533512

[tpj71133-bib-0116] Wang, Z. , Hong, Y. , Zhu, G. , Li, Y. , Niu, Q. , Yao, J. et al. (2020) Loss of salt tolerance during tomato domestication conferred by variation in a Na^+^/K^+^ transporter. EMBO Journal, 39, e103256. Available from: 10.15252/embj.2019103256 PMC7232006 32134151

[tpj71133-bib-0117] Wang, Z. , Zhang, X. , Yang, X. , Tang, H. , Feng, L. , Yin, Y. et al. (2024) Evolution of the *SPX* gene family and its role in the response mechanism to low phosphorus stress in self‐rooted apple stock. BMC Genomics, 25, 488. 10.1186/s12864-024-10402-2 PMC11108120 38755552

[tpj71133-bib-0118] Win, K.T. , Tanaka, F. , Okazaki, K. & Ohwaki, Y. (2018) The ACC deaminase expressing endophyte *Pseudomonas* spp. enhances NaCl stress tolerance by reducing stress‐related ethylene production, resulting in improved growth, photosynthetic performance, and ionic balance in tomato plants. Plant Physiology and Biochemistry, 127, 599–607. 10.1016/j.plaphy.2018.04.038 29730579

[tpj71133-bib-0119] Xiong, L. & Zhu, J.‐K. (2003) Regulation of abscisic acid biosynthesis. Plant Physiology, 133, 29–36. Available from: 10.1104/pp.103.025395 PMC523868 12970472

[tpj71133-bib-0120] Xu, S.‐L. , Rahman, A. , Baskin, T.I. & Kieber, J.J. (2008) Two leucine‐rich repeat receptor kinases mediate signaling, linking cell wall biosynthesis and ACC synthase in Arabidopsis. Plant Cell, 20, 3065–3079. 10.1105/tpc.108.063354 PMC2613664 19017745

[tpj71133-bib-0121] Yang, S.F. (1980) Regulation of ethylene biosynthesis1. HortScience, 15, 238–243.

[tpj71133-bib-0122] Yang, S.F. & Hoffman, N.E. (1984) Ethylene biosynthesis and its regulation in higher plants. Annual Review of Plant Physiology, 35, 155–189.

[tpj71133-bib-0123] Ye, J. , Hu, T. , Yang, C. , Li, H. , Yang, M. , Ijaz, R. et al. (2015) Transcriptome profiling of tomato fruit development reveals transcription factors associated with ascorbic acid, carotenoid and flavonoid biosynthesis. PLoS One, 10, e0130885. 10.1371/journal.pone.0130885 PMC4489915 26133783

[tpj71133-bib-0124] Yin, J. , Zhang, X. , Zhang, G. , Wen, Y. , Liang, G. & Chen, X. (2019) Aminocyclopropane‐1‐carboxylic acid is a key regulator of guard mother cell terminal division in Arabidopsis thaliana. Journal of Experimental Botany, 70, 897–908. 10.1093/jxb/ery413 PMC6363092 30462272

[tpj71133-bib-0125] Zhang, Y. , Zhao, L. , Wang, Y. , Yang, B. & Chen, S. (2008) Enhancement of heavy metal accumulation by tissue specific co‐expression of *iaaM* and *ACC deaminase* genes in plants. Chemosphere, 72, 564–571. 10.1016/j.chemosphere.2008.03.043 18471863

[tpj71133-bib-0126] Zou, Y. , Zhang, Y. & Testerink, C. (2022) Root dynamic growth strategies in response to salinity. Plant, Cell & Environment, 45, 695–704. 10.1111/pce.14205 PMC9298695 34716934

