# Supplementary material for: Root‐sourced 1‐aminocyclopropane‐1‐carboxylic acid modulates development and adaptive responses to salinity in tomato
Source: Plant J. 2026 Oct 8;128(1):e71133. doi: 10.1111/tpj.71133 (PMC13646867; doi:10.1111/tpj.71133)
Supplement: Supplementary file 1 [file TPJ-128-0-s001.pdf]

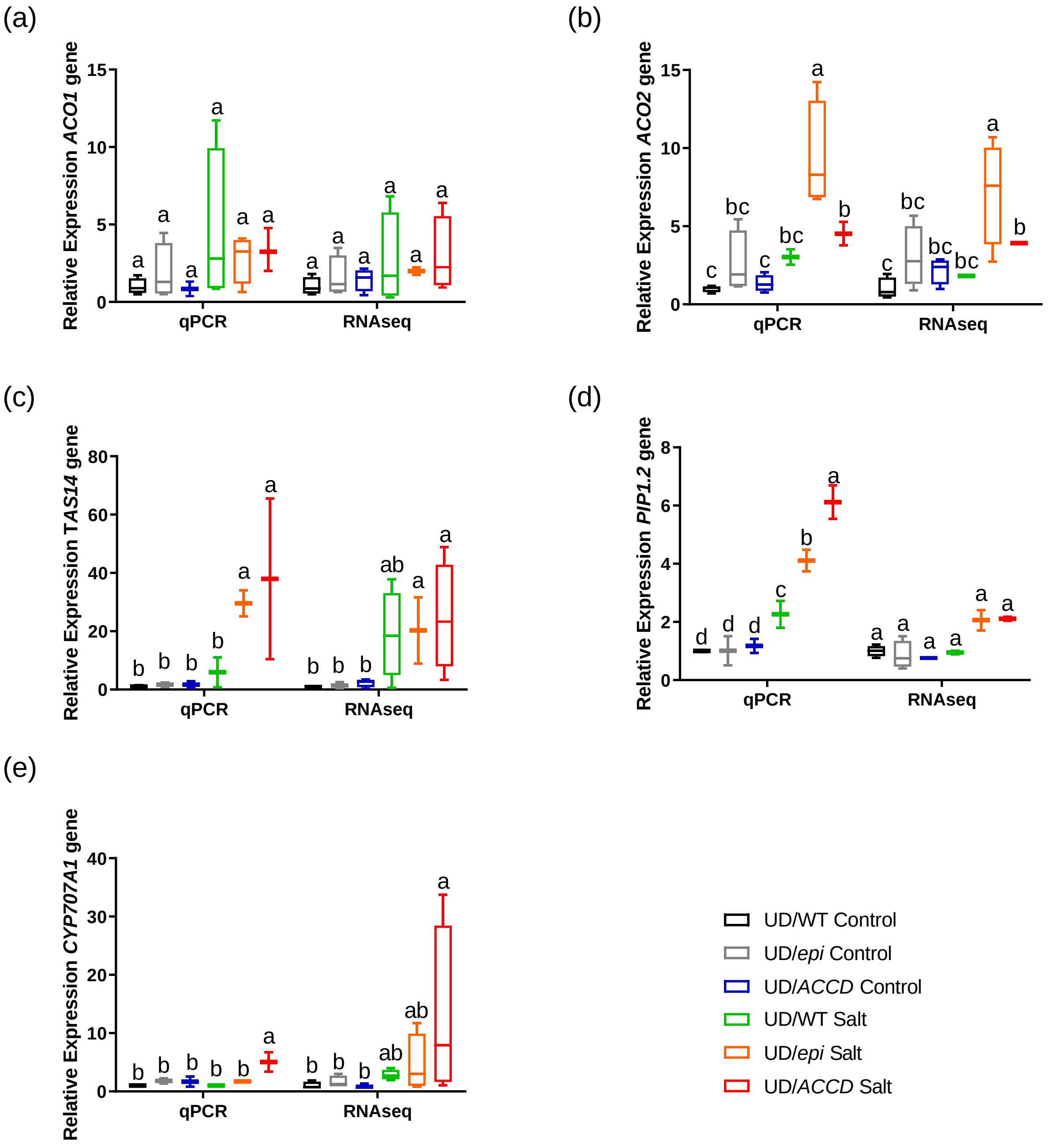
(a)
Relative Expression ACO1 gene
0
5
10
15
qPCR
RNAseq
(b)
Relative Expression ACO2 gene
0
5
10
15
qPCR
RNAseq
(c)
Relative Expression TAS14 gene
0
20
40
60
80
qPCR
RNAseq
(d)
Relative Expression PIP1.2 gene
0
2
4
6
8
qPCR
RNAseq
(e)
Relative Expression CYP707A1 gene
0
10
20
30
40
qPCR
RNAseq
UD/WT Control
UD/epi Control
UD/ACCD Control
UD/WT Salt
UD/epi Salt
UD/ACCD Salt
