# Supplementary material for: Root‐sourced 1‐aminocyclopropane‐1‐carboxylic acid modulates development and adaptive responses to salinity in tomato
Source: Plant J. 2026 Oct 8;128(1):e71133. doi: 10.1111/tpj.71133 (PMC13646867; doi:10.1111/tpj.71133)
Supplement: Supplementary file 2 [file TPJ-128-0-s003.pdf]

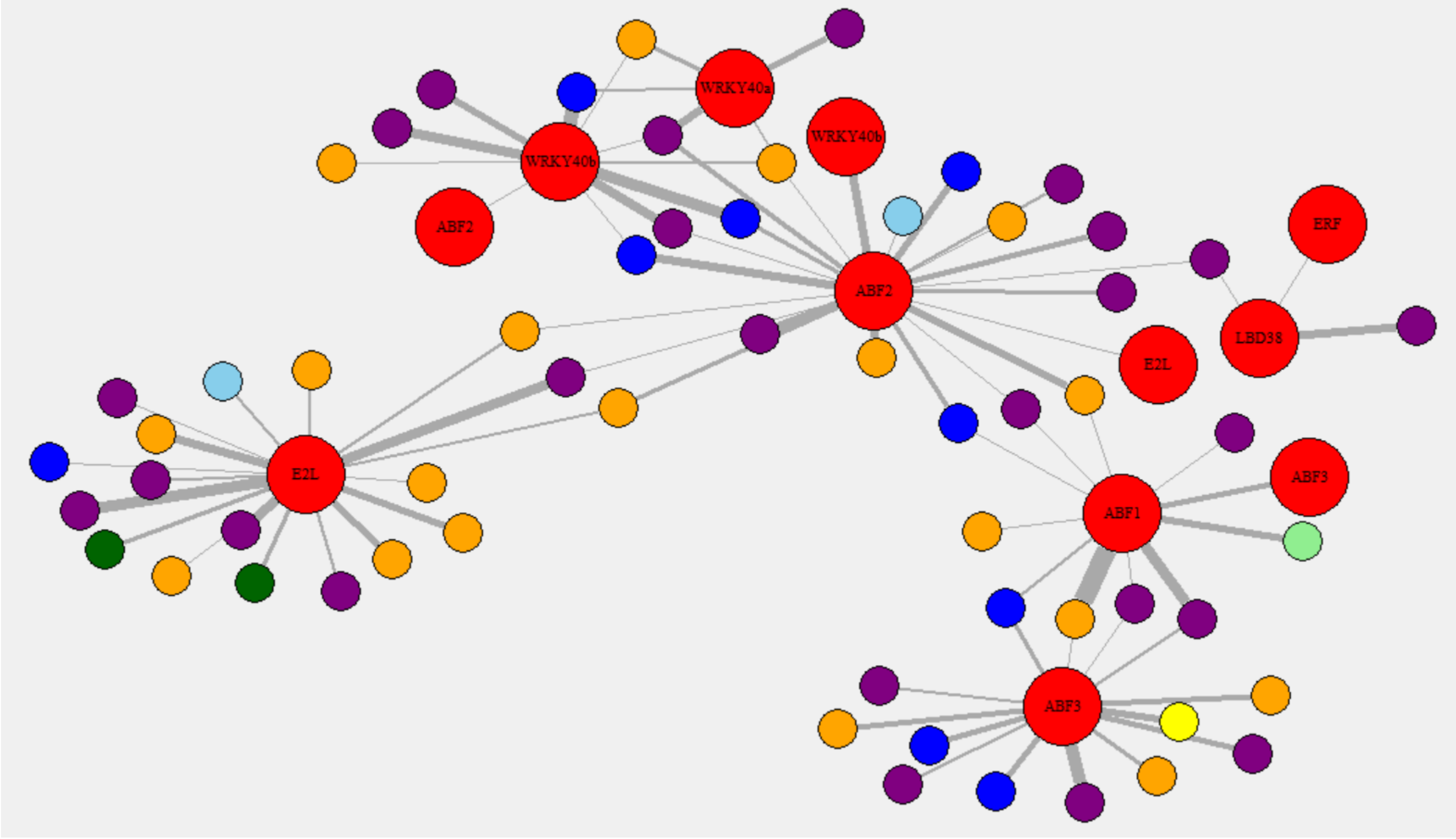

WRKY40a
WRKY40b
WRKY40b
ABF2
ABF2
ERF
LBD38
E2L
E2L
ABF1
ABF3
ABF3
