# Supplementary material for: Root‐sourced 1‐aminocyclopropane‐1‐carboxylic acid modulates development and adaptive responses to salinity in tomato
Source: Plant J. 2026 Oct 8;128(1):e71133. doi: 10.1111/tpj.71133 (PMC13646867; doi:10.1111/tpj.71133)
Supplement: Supplementary file 3 [file TPJ-128-0-s004.pdf]

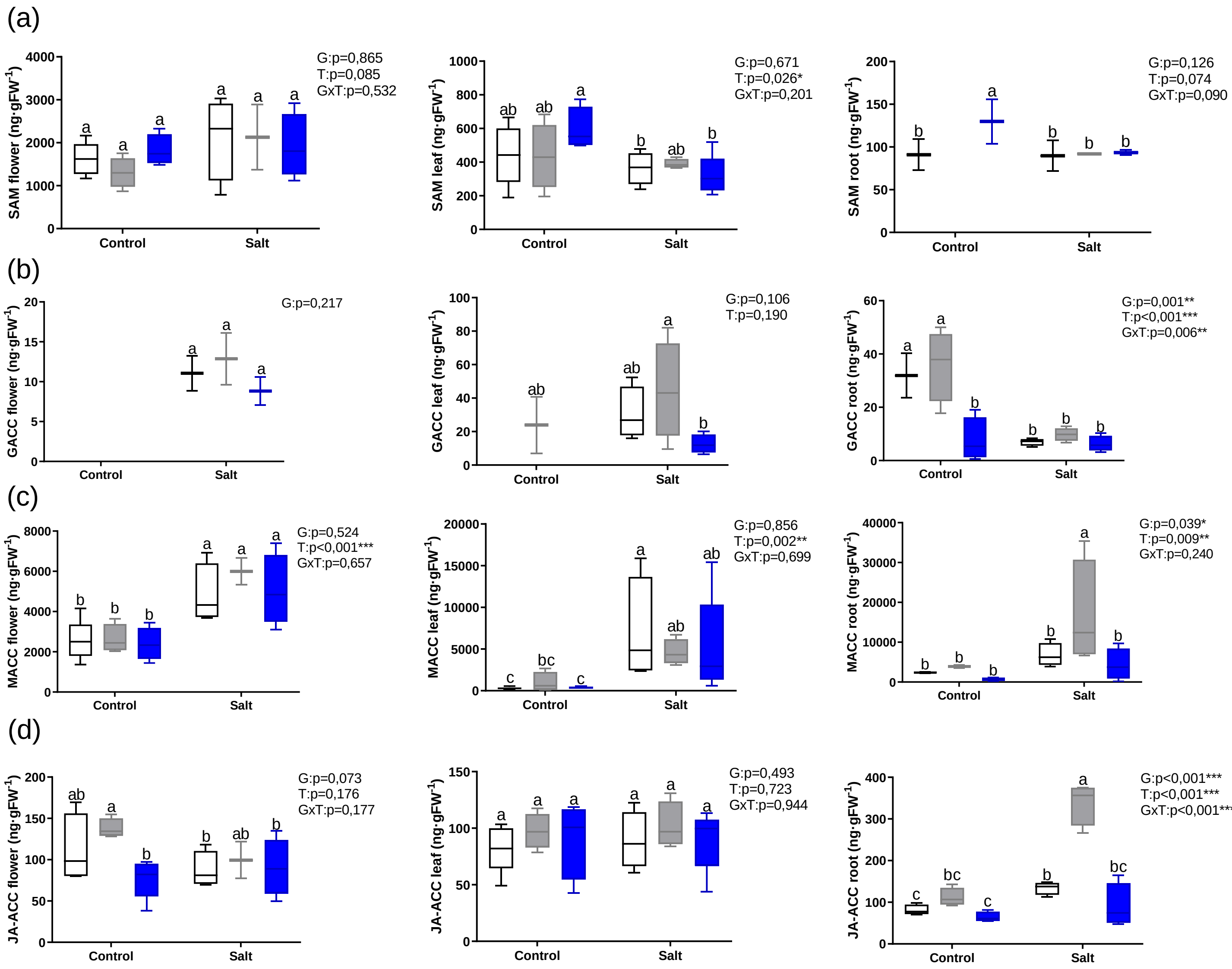
(a)
SAM flower (ng·gFW-1)
G:p=0,865
T:p=0,085
GxT:p=0,532
SAM leaf (ng·gFW-1)
G:p=0,671
T:p=0,026*
GxT:p=0,201
SAM root (ng·gFW-1)
G:p=0,126
T:p=0,074
GxT:p=0,090
(b)
GACC flower (ng·gFW-1)
G:p=0,217
GACC leaf (ng·gFW-1)
G:p=0,106
T:p=0,190
GACC root (ng·gFW-1)
G:p=0,001**
T:p<0,001***
GxT:p=0,006**
(c)
MACC flower (ng·gFW-1)
G:p=0,524
T:p<0,001***
GxT:p=0,657
MACC leaf (ng·gFW-1)
G:p=0,856
T:p=0,002**
GxT:p=0,699
MACC root (ng·gFW-1)
G:p=0,039*
T:p=0,009**
GxT:p=0,240
(d)
JA-ACC flower (ng·gFW-1)
G:p=0,073
T:p=0,176
GxT:p=0,177
JA-ACC leaf (ng·gFW-1)
G:p=0,493
T:p=0,723
GxT:p=0,944
JA-ACC root (ng·gFW-1)
G:p<0,001***
T:p<0,001***
GxT:p<0,001***
Control
Salt
