# Supplementary material for: Root‐sourced 1‐aminocyclopropane‐1‐carboxylic acid modulates development and adaptive responses to salinity in tomato
Source: Plant J. 2026 Oct 8;128(1):e71133. doi: 10.1111/tpj.71133 (PMC13646867; doi:10.1111/tpj.71133)
Supplement: Supplementary file 4 [file TPJ-128-0-s005.pdf]

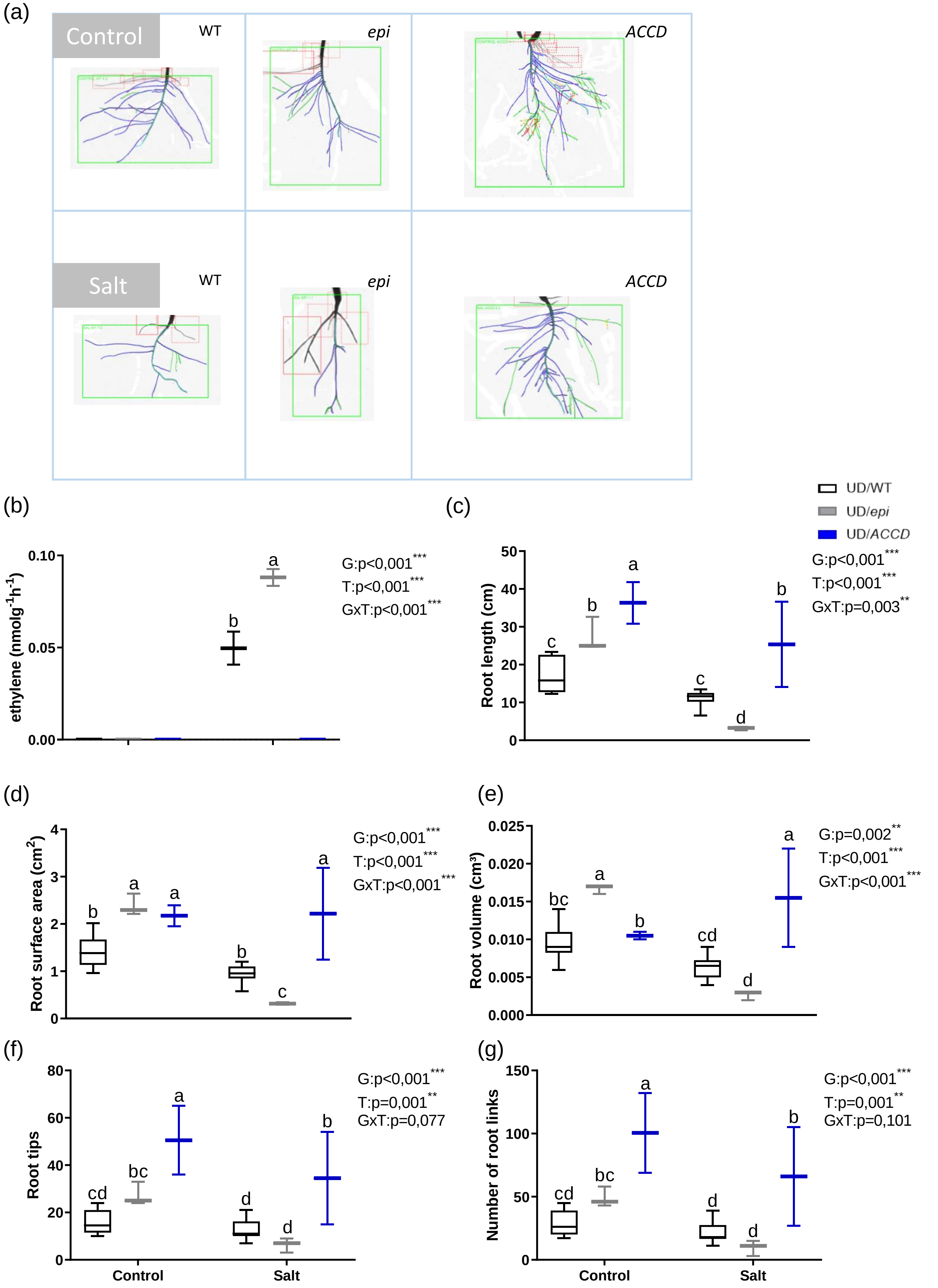

(a)
Control
WT
epi
ACCD
Salt
WT
epi
ACCD
(b)
ethylene (nmolg-1h-1)
0.10
0.05
0.00
a
b
G:p<0,001***
T:p<0,001***
GxT:p<0,001***
(c)
UD/WT
UD/epi
UD/ACCD
Root length (cm)
50
40
30
20
10
0
G:p<0,001***
T:p<0,001***
GxT:p=0,003**
(d)
Root surface area (cm2)
G:p<0,001***
T:p<0,001***
GxT:p<0,001***
(e)
Root volume (cm3)
0.025
0.020
0.015
0.010
0.005
0.000
G:p=0,002**
T:p<0,001***
GxT:p<0,001***
(f)
Root tips
80
60
40
20
0
G:p<0,001***
T:p=0,001**
GxT:p=0,077
Control
Salt
(g)
Number of root links
150
100
50
0
G:p<0,001***
T:p=0,001**
GxT:p=0,101
Control
Salt
