# Supplementary material for: Root‐sourced 1‐aminocyclopropane‐1‐carboxylic acid modulates development and adaptive responses to salinity in tomato
Source: Plant J. 2026 Oct 8;128(1):e71133. doi: 10.1111/tpj.71133 (PMC13646867; doi:10.1111/tpj.71133)
Supplement: Supplementary file 5 [file TPJ-128-0-s006.pdf]

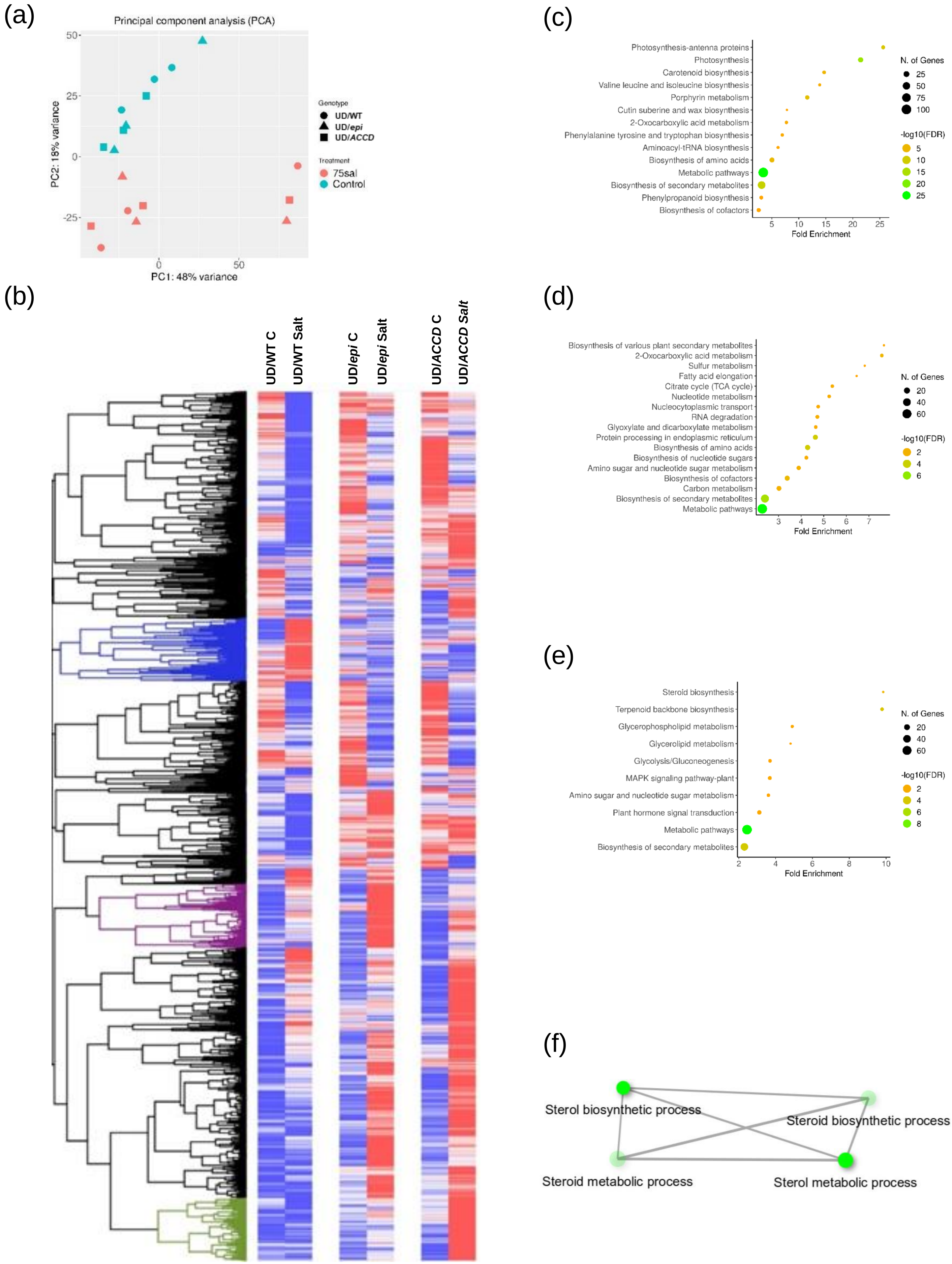

(a)
Principal component analysis (PCA)
PC1: 48% variance
PC2: 18% variance
Genotype
UD/WT
UD/*epi*
UD/*ACCD*
Treatment
75sal
Control
(b)
UD/WT C
UD/WT Salt
UD/*epi* C
UD/*epi* Salt
UD/*ACCD* C
UD/*ACCD* Salt
(c)
Photosynthesis-antenna proteins
Photosynthesis
Carotenoid biosynthesis
Valine leucine and isoleucine biosynthesis
Porphyrin metabolism
Cutin suberine and wax biosynthesis
2-Oxocarboxylic acid metabolism
Phenylalanine tyrosine and tryptophan biosynthesis
Aminoacyl-tRNA biosynthesis
Biosynthesis of amino acids
Metabolic pathways
Biosynthesis of secondary metabolites
Phenylpropanoid biosynthesis
Biosynthesis of cofactors
Fold Enrichment
N. of Genes
-log10(FDR)
(d)
Biosynthesis of various plant secondary metabolites
2-Oxocarboxylic acid metabolism
Sulfur metabolism
Fatty acid elongation
Citrate cycle (TCA cycle)
Nucleotide metabolism
Nucleocytoplasmic transport
RNA degradation
Glyoxylate and dicarboxylate metabolism
Protein processing in endoplasmic reticulum
Biosynthesis of amino acids
Biosynthesis of nucleotide sugars
Amino sugar and nucleotide sugar metabolism
Biosynthesis of cofactors
Carbon metabolism
Biosynthesis of secondary metabolites
Metabolic pathways
Fold Enrichment
N. of Genes
-log10(FDR)
(e)
Steroid biosynthesis
Terpenoid backbone biosynthesis
Glycerophospholipid metabolism
Glycerolipid metabolism
Glycolysis/Gluconeogenesis
MAPK signaling pathway-plant
Amino sugar and nucleotide sugar metabolism
Plant hormone signal transduction
Metabolic pathways
Biosynthesis of secondary metabolites
Fold Enrichment
N. of Genes
-log10(FDR)
(f)
Sterol biosynthetic process
Steroid biosynthetic process
Steroid metabolic process
Sterol metabolic process
