# Supplementary material for: Root‐sourced 1‐aminocyclopropane‐1‐carboxylic acid modulates development and adaptive responses to salinity in tomato
Source: Plant J. 2026 Oct 8;128(1):e71133. doi: 10.1111/tpj.71133 (PMC13646867; doi:10.1111/tpj.71133)
Supplement: Supplementary file 6 [file TPJ-128-0-s007.pdf]

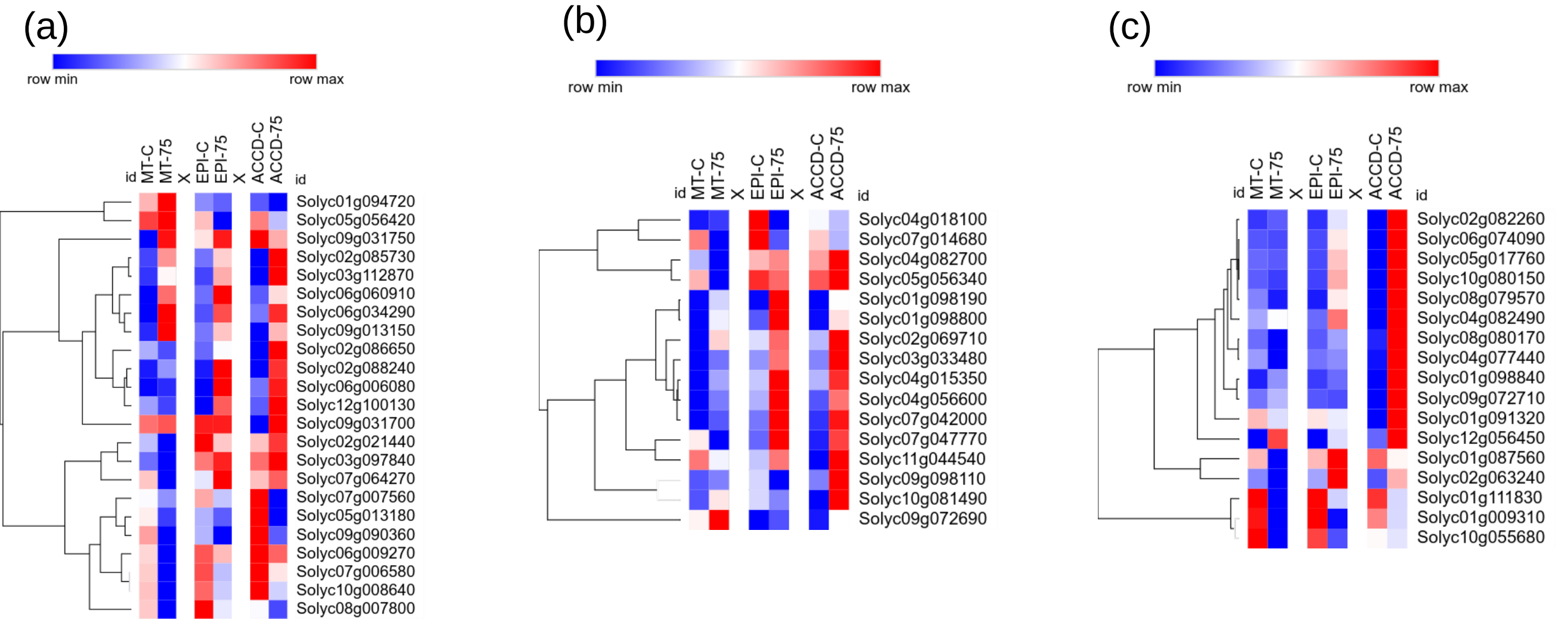

(a)
row min
row max
id
MT-C
MT-75
X
EPI-C
EPI-75
X
ACCD-C
ACCD-75
id
Solyc01g094720
Solyc05g056420
Solyc09g031750
Solyc02g085730
Solyc03g112870
Solyc06g060910
Solyc06g034290
Solyc09g013150
Solyc02g086650
Solyc02g088240
Solyc06g006080
Solyc12g100130
Solyc09g031700
Solyc02g021440
Solyc03g097840
Solyc07g064270
Solyc07g007560
Solyc05g013180
Solyc09g090360
Solyc06g009270
Solyc07g006580
Solyc10g008640
Solyc08g007800
(b)
row min
row max
id
MT-C
MT-75
X
EPI-C
EPI-75
X
ACCD-C
ACCD-75
id
Solyc04g018100
Solyc07g014680
Solyc04g082700
Solyc05g056340
Solyc01g098190
Solyc01g098800
Solyc02g069710
Solyc03g033480
Solyc04g015350
Solyc04g056600
Solyc07g042000
Solyc07g047770
Solyc11g044540
Solyc09g098110
Solyc10g081490
Solyc09g072690
(c)
row min
row max
id
MT-C
MT-75
X
EPI-C
EPI-75
X
ACCD-C
ACCD-75
id
Solyc02g082260
Solyc06g074090
Solyc05g017760
Solyc10g080150
Solyc08g079570
Solyc04g082490
Solyc08g080170
Solyc04g077440
Solyc01g098840
Solyc09g072710
Solyc01g091320
Solyc12g056450
Solyc01g087560
Solyc02g063240
Solyc01g111830
Solyc01g009310
Solyc10g055680
